# How Can Drug Metabolism and Transporter Genetics Inform Psychotropic Prescribing?

**DOI:** 10.3389/fgene.2020.491895

**Published:** 2020-12-08

**Authors:** Beatriz Carvalho Henriques, Esther H. Yang, Diego Lapetina, Michael S. Carr, Vasyl Yavorskyy, Joshua Hague, Katherine J. Aitchison

**Affiliations:** ^1^Department of Psychiatry, University of Alberta, Edmonton, AB, Canada; ^2^Department of Medical Genetics, University of Alberta, Edmonton, AB, Canada; ^3^Neuroscience and Mental Health Institute, University of Alberta, Edmonton, AB, Canada

**Keywords:** pharmacogenomics (PGx), drug metabolism, drug transporters, cytochrome P450 enzymes, psychotropic drugs

## Abstract

Many genetic variants in drug metabolizing enzymes and transporters have been shown to be relevant for treating psychiatric disorders. Associations are strong enough to feature on drug labels and for prescribing guidelines based on such data. A range of commercial tests are available; however, there is variability in included genetic variants, methodology, and interpretation. We herein provide relevant background for understanding clinical associations with specific variants, other factors that are relevant to consider when interpreting such data (such as age, gender, drug–drug interactions), and summarize the data relevant to clinical utility of pharmacogenetic testing in psychiatry and the available prescribing guidelines. We also highlight areas for future research focus in this field.

## Introduction

Genome-wide association studies (GWAS) and related multi-omic strategies lend themselves well to phenotypes with polygenic modes of inheritance. By contrast, pharmacokinetic genes are associated with traits relevant to response to treatment (such as concentrations of medications and their metabolites) in an oligogenic manner with Mendelian patterns of inheritance and relatively large effect sizes. Many of the genes exhibiting a strength of association strong enough for consensus prescribing recommendations are in drug metabolizing enzymes (CPIC; DPWG; Hiemke et al., 2018; PharmGKB; FDA labels). We herein provide a review of the genetics of drug metabolizing enzymes and transporters relevant for medications prescribed in psychiatry. We searched databases such as PubMed, PharmVar, PharmGKB, CPIC, DPWG, and DrugBank as well as relevant reviews, book chapters, and dissertations with search terms including each drug and drug metabolizing enzyme or transporter; each paper thus retrieved was reviewed by a minimum of two coauthors.

Drug metabolism and transport includes Phase I (addition of a reactive group to the molecule), Phase II (transfer of a polar group to the Phase I metabolite), and Phase III (transport of compounds away from the interior of the cells in an energy-dependent manner, introduced by Ishikawa (1992) (Xu et al., 2005). While the liver and gut are most relevant to phase I metabolism, the above activities occur throughout the body, with many drug metabolizing enzymes being widely expressed (Aitchison et al., 2010). Their activity is subject to mechanisms including competitive and non-competitive inhibition, and induction (Pelkonen et al., 2008; Hisaka et al., 2010; Chen F. et al., 2018; Chen J.T. et al., 2018).

## Phase I Metabolism

Phase I enzymes catalyze reactions that alter the hydrophobicity, molecular weight, and reactivity of the substrate, occurring through hydrolysis, reduction, and oxidation reactions. Phase I enzymes relevant to psychotropics include the cytochrome P450 (CYP) family of enzymes, flavin-containing monooxygenases, esterases, epoxide hydrolases (EH), and microsomal epoxide hydrolases (mEH).

## The Cytochrome P450 System

The CYP superfamily and flavin-containing monooxygenases (FMOs) are oxidoreductases. The most studied of these are the CYPs. Individuals sensitive to the antihypertensive agent debrisoquine and to the anti-arrhythmic agent sparteine gave rise to initial observations regarding variable enzyme activity (Mahgoub et al., 1977; Eichelbaum, 1984; Smith, 1986; reviewed in Johansson and Ingelman-Sundberg, 2011). This led to sequencing efforts that identified the first *CYP2D6* loss-of-function mutation (Gough et al., 1990; Hanioka et al., 1990; Kagimoto et al., 1990). Multiple mutations in P450s relevant to psychotropics have since been discovered, with the frequency thereof differing by ethnicity (Aitchison et al., 2000c).

Cytochrome P450 enzyme classification (previously led by the Cytochrome P450 Nomenclature Committee and now transferred to the Pharmacogene Variation Consortium) is as follows: after the letters “CYP” comes a number indicating the CYP family (Cupp and Tracy, 1998). Enzymes within the same family (e.g., CYP1) have a minimum of 36% amino acid sequence homology. The next layer of differentiation is represented by a letter indicating the sub family. Within a subfamily (e.g., CYP1A), there is approximately 70% amino acid homology (Nebert et al., 1987). The final layer is another number representing the isoform (e.g., CYP1A1 and CYP1A2). For all, the enzymes are not italicized, while the corresponding gene names are.

Mutations in the *CYP* genes can be classified in two different manners: pharmacologically in terms of enzyme function, or genetically in terms such as loss or gain of function (van der Weide and Steijns, 1999). Many CYPs have four distinct levels of enzyme activity: poor, intermediate, normal (previously known as extensive), and ultrarapid (Blake et al., 2013; Caudle et al., 2020). Reduced enzyme–substrate affinity, enzyme stability, or splice site variants leading to lack of functional protein can be a result of mutations (for reviews, see, for example, Ingelman-Sundberg, 2004a, b). Gene duplication (or multiple copies, i.e., multiplication) or single nucleotide polymorphisms (SNPs) affecting transcription, on the other hand, may be associated with increased enzyme activity (ultrarapid metabolizers; UM) (Johansson et al., 1993; Sim et al., 2006; Wang et al., 2014, 2015). Gain-of-function variants may increase medication clearance, consequently reducing the concentration, while loss-of-function mutations reduce clearance, increasing the concentration (Zanger and Schwab, 2013); the opposite is true for prodrugs such as codeine, where loss-of-function mutations lead to lack of production of the pharmacologically active analgesic.

### The *CYP1* Family

The CYP1 family includes CYP1A and CYP1B. *CYP1A1* and *CYP1A2* lie in a head–head configuration on chromosome 15, and share a promoter region to which the aryl hydrocarbon receptor binds (at xenobiotic responsive elements) (Jorge-Nebert et al., 2010).

### *CYP1A2* Subfamily

Substrates specific to psychiatric conditions include antipsychotics [e.g., chlorpromazine (CPZ), trifluoperazine, clozapine, olanzapine], tertiary amine tricyclics (e.g., amitriptyline, imipramine, and clomipramine) as well as some selective serotonin reuptake inhibitors (such as fluvoxamine), and zopiclone. Additional substrates include analgesics (paracetamol), anti-inflammatories, cardiovascular agents (e.g., lignocaine), xanthines (caffeine, theophylline, aminophylline), and tacrine (Imaoka and Funae, 1990; Aitchison et al., 2000a, c; Lobo et al., 2008; Turpeinen et al., 2009; Theophylline Pathway; Zanger and Schwab, 2013). CYP1A2 is also involved in toxicity (e.g., bioactivation of arylamines and heterocyclic amines implicated in the formation of colon and bladder cancer, and the neurotoxin 1-methyl-4-phenyl-1,2,3,6-tetrahydropyridine, also known as MPTP) (McManus et al., 1990; Boobis et al., 1994; Eaton et al., 1995; Coleman et al., 1996; Hammons et al., 1997).

The enzyme is inducible by paracetamol, omeprazole, primaquine, carbamazepine, polycyclic aromatic hydrocarbons (e.g., 3-methylcholanthrene), heterocyclic aromatic hydrocarbons (such as 2,3,7,8-tetrachlorodibenzo-p-dioxin), and products of combustion such as cigarette or cannabis smoke (Rost et al., 1994; Parker et al., 1998; reviewed in Aitchison et al., 2000c). Interestingly, a group of phenothiazines represented by perazine and promazine has been shown to induce this enzyme as well, accelerating their own metabolism and that of concomitant medications metabolized by this route (Wójcikowski et al., 2012). It can also be induced by various dietary substances including cruciferous vegetables (*Cruciferae*: including broccoli, brussels sprouts, cabbage, cauliflower, radishes, and watercress), heterocyclic amines (produced in meat browned at high temperatures), and caffeine (Rost et al., 1994; Parker et al., 1998; Aitchison et al., 2000a, c; Ghotbi et al., 2007; Yoshinari et al., 2008; Dobrinas et al., 2011; Arici and Özhan, 2017). Amine metabolism may be affected by cruciferous vegetables for a significant duration (Murray et al., 2001), which may also affect other enzymes. The inducibility by smoking is of particular relevance to psychiatry, as many patients are smokers (discussed in Fiore et al., 1995; Nakajima et al., 1999; Aitchison et al., 2000c; Ghotbi et al., 2007; Dobrinas et al., 2011). Indeed, a recent study conducted by Lesche et al. (2020) on the impact of genotype of various CYP enzymes (including CYP1A2) and the presence of known inducers and inhibitors demonstrated that, for patients prescribed clozapine, a greater percentage of the variation in plasma concentration of this medication was explained by smoking status than by *CYP1A2* genotyping information (in a cohort where 82% of individuals tested positive for the *CYP1A2^∗^1F* variant).

Several polymorphisms have been detected in *CYP1A2* (Jiang et al., 2005; Browning et al., 2010). Individuals with the *CYP1A2^∗^1F* c.−163C>A SNP that confers higher inducibility (Sachse et al., 1998; Chida et al., 1999; Han et al., 2002; Sim, 2013) have higher levels of caffeine metabolism. An initial report in Whites showed a reduction in olanzapine serum concentration in association with this variant (Laika et al., 2010). A subsequent study in Norwegian Whites was not able to replicate this association with olanzapine serum concentration, but in CSF, the ratio of 4’-*N*-desmethylolanzapine to olanzapine was associated with smoking and *CYP1A2* genotype, with the highest ratios being in smokers homozygous for the *CYP1A2^∗^1F* (Skogh et al., 2011). A later paper by the same group was also not able to replicate an association between the *CYP1A2^∗^1F* and systemic exposure to olanzapine, but did find a relatively modest effect of other variants (rs2472297C>T lying in the intergenic region between *CYP1A1* and *CYP1A2* and rs4410790C>T upstream of the aryl hydrocarbon receptor locus; Söderberg et al., 2013). Attempts at replication of an association between the *CYP1A2^∗^1F* and olanzapine exposure in Asians have also been negative (Shimoda et al., 2002; Obase et al., 2003; Chen et al., 2005; Ghotbi et al., 2007; Nozawa et al., 2008).

There are also loss-of-function variants. The *CYP1A2^∗^6* haplotype containing the c.1291 C>T (previously known as the c.5090 C>T) mutation causes an Arg431Trp amino acid substitution resulting in a complete loss of enzyme function (Chevalier et al., 2001; Zhou et al., 2004). By analogy, owing to the behavioral effects seen on administering clozapine to a CYP1A2 knockout mouse, it is possible that people with this variant could experience more side effects of medications metabolized by CYP1A2, including clozapine and olanzapine (Aitchison et al., 2000b). The *CYP1A2^∗^1C* haplotype has a promoter mutation (−3860 G>A) which has been associated with a reduction in caffeine metabolism in Japanese (Nakajima et al., 1999). Other known variants of *CYP1A2* with decreased activity include *CYP1A2^∗^1K* (characterized by polymorphisms −729C>T, −739T>G, and −163C>A), identified in an Ethiopian population (Aklillu et al., 2003). Likewise, variants *CYP1A2^∗^3* (2116 G>A and 5347 T>C) and *CYP1A2^∗^4* (2499 A>T) are associated with reduced activity and have been identified (Chevalier et al., 2001; Zhou et al., 2004).^1^ The *CYP1A2^∗^7* has a splice site mutation in the donor site of intron 6 (3533G>A) and was found in heterozygous state in one patient with very high clozapine concentration and plasma caffeine clearance at the lower limit of the normal range, consistent with the mutation leading to no functional CYP1A2 enzyme (Allorge et al., 2003).

Many agents also contribute to the inhibition of CYP1A2, such as: apiaceous vegetables (parsnips, celery, dill, parsley) (Lampe et al., 2000), fluvoxamine (Brosen et al., 1993), grapefruit juice (Fuhr et al., 1993), estrogens (Knutti et al., 1981; Rietveld et al., 1984; Abernethy and Todd, 1985; Vistisen et al., 1992; Le Marchand et al., 1997), quinolone antibiotics (Fuhr et al., 1992), and in smokers, heavy ethanol consumption (Rizzo et al., 1997).

### The *CYP2* Family

*CYP2* genes comprise clusters on different chromosomes (Sezutsu et al., 2013; Zanger and Schwab, 2013).

### *CYP2A* Subfamily

The CYP2A subfamily includes *CYP2A6* and *CYP2A13* (Hoffman et al., 2001; Zanger and Schwab, 2013), with *CYP2A6* being of relevance to psychiatry. CYP2A6 is mainly expressed in the liver, where it accounts for approximately 4% of total CYP content (Shimada et al., 1994; Haberl et al., 2005). CYP2A13 is expressed at reduced levels in the respiratory tract (Leclerc et al., 2011; Raunio and Rahnasto-Rilla, 2012). CYP2A6 was first recognized as the enzyme responsible for coumarin 7-hydroxylation, and is also the primary nicotine *C*-oxidase (Pelkonen et al., 2000; Fuhr et al., 2007; Mwenifumbo et al., 2007; Raunio and Rahnasto-Rilla, 2012). In addition to nicotine, CYP2A6 contributes to the metabolism of promazine, valproic acid, disulfiram, and caffeine as well as to other medications and toxins (Crespi et al., 1990; Yamazaki et al., 1992; Gonzalez and Gelboin, 1994; Oscarson et al., 1998; Komatsu et al., 2000; Murai et al., 2009; Tanner and Tyndale, 2017).

Like many of the CYP superfamily, *CYP2A6* is a highly polymorphic gene, with many known mutations affecting enzymatic activity (Di et al., 2009; McDonagh et al., 2012). Polymorphisms arise from the occurrence of gene conversion events, deletions, duplications, multiple nucleotide insertions/deletions, and SNPs. The frequency of these events varies by ethnicity, with Asians having the highest frequency of loss-of-function mutations (∼50%), and Whites the least (∼9%) (Nakajima et al., 2006; di Iulio et al., 2009). CYP2A6 expression and activity are also impacted by induction and inhibition effects, age, and interactions with other hepatic enzymes, co-enzymes, and co-factors (Tanner and Tyndale, 2017).

Loss-of-function is often a result of the common *CYP2A6^∗^2* and *CYP2A6^∗^4* alleles. With a frequency of 1–5% in Whites, the *CYP2A6^∗^2* rs1801272 SNP encodes an inactive enzyme due to a Leu160His substitution (Tanner et al., 2017). The *CYP2A6^∗^4* haplotype (and its subtypes such as *CYP2A6^∗^4A* and *CYP2A6^∗^4H*) denotes a complete gene deletion, where the subtypes represent different genomic mechanisms for the deletion. This deletion is found at higher frequencies in Asians and Blacks [e.g., *CYP2A6^∗^4* has a haplotype frequency of up to 15% in a specific Asian group (Pang et al., 2015)]. Other variants such as *CYP2A6^∗^9* result in a reduced enzyme functionality. Both complete loss-of-function and reduced function variants may result in a reduction of treatment efficacy, with atypical metabolite formation (e.g., switching from coumarin 7-hydroxylation to 3-hydroxylation) (Hadidi et al., 1997; Komatsu et al., 2000; Fujita and Sasaki, 2007). Associations between CYP2A6 variants and smoking cessation have been reported (reviewed by Tanner and Tyndale, 2017).

### *CYP2B* Subfamily

The *CYP2B* subfamily members are *CYP2B6* and a *CYP2B7P* (a pseudogene). CYP2B6 is strongly induced by phenobarbital (Faucette et al., 2004). It accounts for ∼1% of total hepatic CYP content (Ward et al., 2003), with variance in inter-individual expression of up to 300-fold (Lang et al., 2001; Lamba et al., 2003; Desta et al., 2007; Hofmann et al., 2008; Wang and Tompkins, 2008; Ohtsuki et al., 2012).

There are 38 different *CYP2B6* haplotypes currently described, some of which are associated with defined changes in enzyme function (Lang et al., 2001, 2004; Lamba et al., 2003; Klein et al., 2005; “Pharmacogene Variation Consortium: CYP2B6”; Zukunft et al., 2005). Two of the haplotypes are structural variants representing hybrids whose sequence is partly derived from *CYP2B6* and partly from *CYP2B7P*, and gene duplications have also been identified (Martis et al., 2013). With a frequency of 15–50% across different ethnicities (of which Blacks have the highest), the most common allele is *CYP2B6^∗^6*. The mutations c.516G>A and c.785A>G lead to amino acid substitutions Gln172His and Lys262Arg respectively, and are associated with a reduction in enzyme activity (Lang et al., 2001; Tsuchiya et al., 2004; Hofmann et al., 2008; Turpeinen and Zanger, 2012). Those who contain homozygous copies of *CYP2B6^∗^6* show increased plasma concentrations of relevant drugs, which has been linked to increased risk of Adverse Drug Reactions (ADRs) (Haas et al., 2004; Ribaudo et al., 2006; Zanger et al., 2007; King and Aberg, 2008; Lubomirov et al., 2011; Yimer et al., 2012; Zanger and Klein, 2013). The *CYP2B6^∗^4* has a haplotype frequency averaging at 9%, being up to 45% in Africans, 27% in Hispanics, 21% in Europeans, and 19% in Asians (Reference SNP:rs2279343). The enzyme encoded by this variant clears bupropion [relevant for smoking cessation and also used in the treatment of depression and attention deficit hyperactivity disorder (ADHD)] more rapidly than the wild-type (Lang et al., 2001; Kirchheiner et al., 2003; Hesse et al., 2004; Rotger et al., 2007). The *CYP2B6^∗^18* contains the c.983T>C substitution, which leads to Ile328Thr substitution. This haplotype is found in some African populations (e.g., the Bantu) (Jamshidi et al., 2010).

#### *CYP2C* Subfamily

Forming a ∼390 kb cluster at chromosome 10q24, the *CYP2C* subfamily contains four genes: *CYP2C8, CYP2C9, CYP2C18*, and *CYP2C19* (Goldstein and de Morais, 1994; Nelson et al., 2004). All exhibit extensive homology in both DNA and amino acid sequence, and are thus responsible for the metabolism of partially overlapping subsets of drugs (Coller et al., 2002; Koukouritaki et al., 2004; Rettie and Jones, 2005; Lai et al., 2009; Naraharisetti et al., 2010; Ohtsuki et al., 2012). Their main expression is in the liver, comprising 20% of hepatic CYP content (Shimada et al., 1994). Within the CYP2C subfamily, CYP2C9 is the most abundantly expressed, followed by CYP2C8 and CYP2C19. Psychotropic substrates for CYP2C19 include diazepam (Jung et al., 1997), phenytoin (Bajpai et al., 1996; Mamiya et al., 1998), propranolol (Otton et al., 1990), selective serotonin reuptake inhibitors (SSRIs; Hicks et al., 2015), and tricyclics (Hicks et al., 2017). *CYP2C18* is distal to *CYP2C19* on chromosome 10 but appears to be expressed only at the mRNA level and not at the protein level (Chen and Goldstein, 2009).

CYP2C8 is the second most important cytochrome after CYP3A4 for the conversion of buprenorphine to its active metabolite, norbuprenorphine (Picard et al., 2005), and its expression is under genetic control. Work in Asian populations has identified variants that are associated with no functional enzyme, specifically the *CYP2C8^∗^5.001*, the *CYP2C8^∗^7.001*, and the *CYP2C8^∗^11.001* at 0.006, 0.0025, and 0.003 (in Koreans; 0.01 in Vietnamese and 0.0014 in Chinese) frequency, respectively in E. Asian populations tested (Soyama et al., 2002; Hichiya et al., 2005; Yeo et al., 2011).

CYP2C9 metabolizes phenytoin. It is also relevant to drugs prescribed to treat physical comorbidities in those with chronic mental health conditions. These include anti-diabetic agents (such as tolbutamide, glimepiride, and nateglinide), angiotensin II blockers (losartan, valsartan, candesartan, and irbesartan), fluvastatin, warfarin, and nonsteroidal anti-inflammatory drugs including COX2 inhibitors (e.g., celecoxib) (Michaels and Wang, 2014). Of the reduced function variants, *CYP2C9^∗^2* and *CYP2C9^∗^3* are the most common, and have been studied in relation to the metabolism of drugs with a narrow therapeutic index, such as phenytoin, tolbutamide, and warfarin (Lee et al., 2002). The functional mutations in *CYP2C9^∗^2* and *CYP2C9^∗^3* are rs1799853 and rs1057910, leading to Arg144Cys and Ile259Leu substitutions. *CYP2C9^∗^2* is associated with a 10-fold lower V_*max*_ and 2-fold lower V_*m*_ for (*S*)-warfarin hydroxylation. Median daily warfarin dose was in one study 4.0, 2.9, 2.6, and 1 mg for individuals of *CYP2C9^∗^1/^∗^1*, *CYP2C9^∗^1/^∗^2*, *CYP2C9^∗^1/^∗^3*, and *CYP2C9* homozygous mutant genotype, respectively (King et al., 2004). Individuals who are affected by two reduced function alleles have a greater chance of ADRs such as gastrointestinal bleeding from NSAIDs (Martinez et al., 2004), hypoglycemia (Holstein et al., 2005), and bleeding from warfarin (Ogg et al., 1999). In a GWAS of response to warfarin, a *CYP2C9* marker was separately genotyped in addition to the array-based genomic analysis and was identified as the top signal (Takeuchi et al., 2009). Predictive modeling followed, and included a target of the drug (*VKORC1*), as well as *CYP2C9* (Eriksson and Wadelius, 2012; Maagdenberg et al., 2018); the FDA label summarizes findings of a meta-analysis in which patients carrying at least one copy of the *CYP2C9^∗^2* or *CYP2C9^∗^3* alleles required a mean daily warfarin dose 17 or 37%, respectively less than wild-type individuals (Sanderson et al., 2005).

Like many CYPs (Kalow and Tyndale, 1992), CYP2C19 is also expressed extrahepatically in multiple tissues including in the brain (Aitchison et al., 2010). Substrates of this enzyme include: diazepam and its metabolite desmethyldiazepam, moclobemide (Roh et al., 1996), SSRIs (fluoxetine, sertraline, paroxetine, citalopram, escitalopram), tertiary amine tricyclics (e.g., amitriptyline, imipramine, and clomipramine), as well as clozapine, olanzapine, phenytoin, and propranolol to lesser extents. The SSRIs fluoxetine and fluvoxamine also inhibit CYP2C19 (Preskorn, 1997). Conversely, phenothiazines represented by perazine and promazine have been shown to induce this CYP enzyme (Wójcikowski et al., 2012). Other substrates include the anticoagulant clopidogrel, cyclophosphamide, nelfinavir, proguanil, proton pump inhibitors [omeprazole (Karam et al., 1996) and pantoprazole], thalidomide, and voriconazole (Desta et al., 2002). Of the 35 allelic variants described in the CYP Database, *CYP2C19^∗^2*-*^∗^8* are the most common loss of function (poor metabolizer or PM) haplotypes.

There is substantial interethnic variation in the incidence of PMs of CYP2C19, being 2–5% in Whites, 2% in Saudi Arabians, 4% in Black Zimbabweans, 5% in Ethiopians, 13% in Koreans, 15–17% in Chinese, 21% in Indians, and 18–23% in Japanese (Evans et al., 1995; Aitchison et al., 2000c). When the square root of the PM phenotypic frequency (equal to the frequency of PM *CYP2C19* alleles) is plotted versus longitude, an increase in this value versus longitude may be seen, with an increment in the value occurring between Saudi Arabia and Bombay (Evans et al., 1995; Saeed and Mayet, 2013). The increasing frequency of PMs is mainly owing to the higher frequencies of the null haplotypes *CYP2C19^∗^2* and *CYP2C19^∗^3*. The most common gain-of-function haplotype is the c.−806C>T (rs12248560) defining the *CYP2C19^∗^17* haplotype. Of note, however, this may be found in combination with loss-of-function variants such as the c.1A>G (rs28399504) associated with the *CYP2C19^∗^4* haplotype, or another loss of function variant (c.463G>T) (Scott et al., 2012, 2013; Skierka and Black, 2014). It is therefore necessary to accurately characterize haplotypes with the c.−806C>T. Tables available via PharmGKB^2^ provides further details on *CYP2C19* haplotype frequencies by ethnic group.

The most common PM haplotype is *CYP2C19^∗^2*, which accounts for about 86% of all the PMs in the White population and 69–87% in the E. Asian population. The substitution of G681A in exon 5 of the *CYP2C19^∗^2* haplotype creates an aberrant splice site (de Morais et al., 1994). The second most common PM haplotype is *CYP2C19^∗^3*, which represents about 13–31% of E. Asian PMs and 1.5% of White PMs. The substitution of G636A mutation in exon 4 of the *CYP2C19^∗^3* creates a premature stop codon. A third variant, *CYP2C19^∗^4* accounts for approximately 3% of White PM alleles and contains an A → G mutation in the initiation codon (i.e., c. 1A>G). *CYP2C19^∗^5* accounts for 1.5% of White PM alleles and is rare in E. Asians. The *CYP2C19^∗^5* haplotype is a result of a c.C1297T mutation in exon 9, in which causes an Arg433Trp change in the heme-binding region. *CYP2C19^∗^6* (a c. G395A base substitution resulting in an Arg132Gln coding change in exon 3) and *CYP2C19^∗^7* (a GT → GA mutation in the donor splice site of intron 5 at c.819 +2) each account for a further 1.5% of White PM alleles. *CYP2C19^∗^8*, a T358C substitution in exon 3 that result in a Trp120Arg change, is a less common PM allele. The products of *CYP2C19^∗^6* and *CYP2C19^∗^8* show reduced catalytic activity (2% and 9% of wild-type S-mephenytoin hydroxylase activity, respectively); the others described above are associated with failure to express active CYP2C19. *CYP2C19^∗^2A* and *CYP2C19^∗^3* have both been identified in an Ethiopian population and found to account for all the PM alleles in the 114 individuals studied (Persson et al., 1996).

In CYP2C19 PMs, diazepam clearance is significantly lower than in NMs (Bertilsson, 1995). The mean clearance is lower in Chinese compared to Whites. Owing to the relatively high frequency of PMs in E. Asians, there is a greater frequency of individuals carrying one PM haplotype (i.e., heterozygous PMs). Consistent with this, “many Hong Kong physicians routinely prescribe smaller diazepam doses for Chinese than for white Whites” (Kumana et al., 1987). The main variant responsible for this effect is the G681A, which has a gene-dosage association effect on diazepam clearance (Qin et al., 1999). For recent data on antidepressants and CYP2C19, see the relevant section. Dose adjustment by *CYP2C19* genotype has been published for amitriptyline, citalopram, clomipramine, imipramine, moclobemide, and trimipramine (Kirchheiner et al., 2001). CYP2C19 PMs show a significantly higher efficacy for triple therapy for *Helicobacter pylori* (proton pump inhibitor, clarithromycin, and amoxicillin) (Klotz, 2006, 2009).

#### *CYP2D* Subfamily

The *CYP2D* subfamily consists of a gene cluster comprising *CYP2D6*, with two pseudogenes, *CYP2D7* and *CYP2D8* (Yasukochi and Satta, 2011). CYP2D6 accounts for 1.5% of microsomal CYP content in the liver (Michaels and Wang, 2014), and is involved in metabolizing the majority of psychotropic drugs (Bertilsson et al., 2002). It is also expressed in other organs, including the brain (Niznik et al., 1990; Kalow and Tyndale, 1992; Siegle et al., 2001; Aitchison et al., 2010), and has been associated with synthesis of neurotransmitters (Yu et al., 2003; Niwa et al., 2017). The *CYP2D6* gene is highly polymorphic, even compared to some of the other CYPs (Pharmacogene Variation Consortium: CYP2D6). It is the most extensively studied genetically variable drug metabolizing enzyme (Bertilsson et al., 2002; Ingelman-Sundberg, 2004b), and has over 110 unique alleles identified (Kertesz et al., 2007).

These studies have revealed that there is significant variation of allelic variants between ethnic groups (Aitchison et al., 2000c)^3^. For example, the *CYP2D6^∗^4* haplotype (previously known as g.1846G>A, genomic location of NG_008376.3 (Reference SNP (refSNP) Cluster Report: rs3892097) 1847G>A, Gough et al., 1990; Hanioka et al., 1990; Kagimoto et al., 1990) has a frequency of 19% in Whites (approximately 70–90% of all the PM alleles) (Aitchison et al., 1999), and 6% in Africans and 1% in South Asians (Aitchison et al., 2000c; Mammen et al., 2018). The second most frequent PM haplotype in Whites (2–2.5%) is *CYP2D6^∗^5* (Aitchison et al., 1999), which represents a complete gene deletion, and occurs at a frequency of 5.3, 2.9, and 2.9% in Africans, Asians, and Hispanics, respectively (Bradford, 2002; Del Tredici et al., 2018). The *CYP2D6^∗^10* haplotype has key C188T and G4268C base substitutions in exons 1 and 9, respectively, that result in Pro34Ser and Ser486Thr amino acid substitutions (Yokota et al., 1993; Sakuyama et al., 2008). This haplotype is associated with reduced enzymatic activity (Johansson et al., 1994). With an allelic frequency of 0.43, it is very high in East Asians (Aitchison et al., 2000c; Mammen et al., 2018), and similar to other reduced activity metabolizers has been associated with ADRs such as tardive dyskinesia (Ohmori et al., 1998; Puangpetch et al., 2016). However, some of these apparent *CYP2D6^∗^10* alleles may in fact be *CYP2D6^∗^36* hybrid alleles. The *CYP2D6^∗^17* haplotype exhibits a similar reduction in enzymatic activity (Masimirembwa et al., 1996; Oscarson et al., 1997), and is found predominantly in Africans, with frequencies of 34% in Zimbabwe, 28% in Ghana, 17% in Tanzania, and 9% in Ethiopia (Bertilsson et al., 2002). *CYP2D6^∗^41* is the most common reduced activity (IM) haplotype in Whites, a key SNP 2989G>A (genomic position on NG_008376.3 7189G>A) occupying an intronic position leading to a splicing defect (Raimundo et al., 2000, 2004; Rau et al., 2006; Toscano et al., 2006; Hicks et al., 2013, 2016; Wang et al., 2014). Tables available via PharmGKB^4^ provides details of *CYP2D6* haplotype frequencies by ethnic group.

At the opposite end of the activity spectrum are the UM allelic variants, which most commonly have extra functional copies of the *CYP2D6* gene in tandem on the chromosome, seen at a frequency of 0.9–4% in Whites (Johansson et al., 1993; Aitchison et al., 1999). An apparently less common mechanism for UM alleles is upregulation of gene expression owing to SNP-related enhancer activity (Wang et al., 2015). Individuals possessing UM alleles were first identified as having lower than expected blood concentration of tricyclic antidepressants such as clomipramine (Bertilsson et al., 1993a, b; Dalen et al., 1998; Roberts et al., 2004). *CYP2D6* gene duplication or multiplication events occur at rates up to 29% in Ethiopians (Aklillu et al., 1996) by old techniques such as restriction fragment length polymorphism, and remain to be accurately characterized in terms of frequency using more current approaches.

The diversity in *CYP2D6* phenotype has clinical implications (Ingelman-Sundberg, 2005). Individuals with two PM haplotypes have no functional enzyme, are classified as PMs, and are more prone to ADRs for drugs with a narrow therapeutic window (Steimer et al., 2004, 2005). At the other end of the spectrum, UMs may also show more ADRs, such as tardive dyskinesia (Koola et al., 2014) or symptoms of morphine overdose on codeine (Crews et al., 2014), owing to enhanced formation of toxic metabolites (Pinto and Dolan, 2011). Variation in *CYP2D6* is highly relevant to psychiatry: for most antidepressants and antipsychotics, there are clinical guidelines that state that pharmacogenomic information for *CYP2D6* could or should be used in prescribing (Bousman et al., 2019a). A review with modeling found that for antidepressants metabolized by CYP2D6, normal metabolizers (NMs) would require at least double the dose required by PMs, while cost analyses have associated PM status with not only higher ADRs but also with more drop outs from treatment (Chou et al., 2003; Kirchheiner et al., 2004; Zhou et al., 2004; Tanner et al., 2020).

Haplotype functionality may be used to derive an activity score (Caudle et al., 2020), with resources provided by PharmGKB to assist with this process.^3^ In the most recent update, the activity score of the *CYP2D6^∗^10* haplotype was adjusted from 0.5 to 0.25, and the phenotype assignment for an activity score of 1 adjusted from NM to IM.

#### *CYP2E* Subfamily

A relatively small number of allelic variants have been identified for *CYP2E1*, such as *CYP2E1^∗^2*, which is associated with reduced enzyme activity (Hu et al., 1997; Mittal et al., 2015). This enzyme is produced primarily in the liver, although it is also found in the brain (García-Suástegui et al., 2017), and is responsible for metabolizing ethanol (into acetaldehyde), paracetamol/acetaminophen, and other substances into reactive intermediates, whose toxicity is enhanced in alcoholics (Cederbaum, 2012). Indeed, CYP2E1 is responsible for 20% of total ethanol metabolism (to which other enzymes such as catalases also contribute) (Heit et al., 2013). Gene transcription is induced by ethanol consumption (a moderate level of intake at 140 g ethanol per week producing an increase in expression of CYP2E1 in the intestine, but not in the liver; Liangpunsakul et al., 2005). Interestingly, a 96-bp insertion polymorphism in the *CYP2E1* gene, which is associated with higher activity of the encoded enzyme, has been proposed as a possible protective factor against alcoholism (Cartmell et al., 2005). In addition to its relevance to alcohol use disorders, the role of CYP2E1 in metabolizing ethanol is a potential alcohol–drug interaction site. With occasional alcohol usage, medications such as clozapine at least partly metabolized by CYP2E1 may have their half-life increased owing to competitive inhibition with alcohol. With chronic alcohol use, the induction effect predominates, thus reducing the efficacy of CYP2E1-dependent drugs by decreasing half-life (Cederbaum, 2012).

In mice, tobacco smoke induces CYP2E1 activity in the lungs, liver and kidney (Zevin and Benowitz, 1999). In male smokers, CYPE1 clearance may be increased (Benowitz et al., 1999). There may additionally be a complicated interaction effect of smoking and alcohol at CYP2E1, whereby CYP2E1 activity (as measured by chlorzoxazone metabolism) appears to be enhanced in non-alcoholic female smokers (Girre et al., 1994), while in males (Howard et al., 2003) *CYP2E1^∗^1D* has been associated with nicotine and alcohol co-dependence in one study (Howard et al., 2003), which was not replicated in Taiwanese (Huang et al., 2018).

### The *CYP3* family

The CYP3 family comprises the *CYP3A* subfamily of four genes (*CYP3A4*, *CYP3A5*, *CYP3A7*, and *CYP3A43*) and two pseudogenes (*CYP3AP1* and *CYP3AP2*). CYP3A4 is the most abundant, although CYP3A4 and CYP3A5 have overlapping substrate specificity and in those deficient in CYP3A4, CYP3A5 and other members of the CYP3A family become crucial. The sum of the activity of all CYP3As is the total CYP3A activity, which is responsible for metabolizing ∼50% of all clinically relevant drugs (Guengerich, 1999; Bu, 2006) as well as endogenous and exogenous steroids. They are found mainly in the liver, with lower concentrations found in the intestine, respiratory tract, brain, lung and kidney (Shimada et al., 1994). Owing to their intestinal and hepatic locations, these enzymes play a significant role in the first pass metabolism of all orally administered drugs. Similar substrate specificity is due to high sequence similarity between the enzymes. CYP3A can exhibit substantial interindividual and interethnic variation in its enzymatic activity or expression, partly owing to genetic polymorphism, marked effects of inducers and inhibitors, and epigenetic mechanisms of regulation of gene expression. CYP3A inducers (such as carbamazepine, phenytoin, rifampicin, and phenothiazines such as perazine and promazine) can greatly decrease plasma concentrations of other CYP3A substrates, resulting in reduced efficacy of the substrate (Wójcikowski et al., 2012; Gupta et al., 2018). Conversely, the administration of CYP3A inhibitors (e.g., ketoconazole) can increase the plasma concentration of other substrates, increasing ADRs or even toxicity.

Inhibition/induction effects at the level of the intestine may be more important than those occurring at the hepatic level for certain drugs in some individuals. Indeed, the effects of efflux transporters such as p-glycoprotein can increase exposure of drugs to CYP3A enzymes in the intestine by prolonging transit time across the enterocyte (Wacher et al., 2001). Interestingly, there is broad overlap between substrates for and inhibitors of CYP3A enzymes and p-glycoprotein (Bruyere et al., 2010).

CYP3A4 is the most abundant CYP3A isoform in the intestine and liver (Nebert and Russell, 2002). Up to 30-fold interindividual variation in activity is seen (Ma et al., 2002); however, unlike the distribution of enzymes strongly under genetic control (such as CYP2D6), the distribution is unimodal. Some functional polymorphisms, such as *CYP3A4^∗^22* (a intron 6 SNP, rs35599367, C>T), which is a loss of function mutation associated with 1.7–2.5 decrease in mRNA expression for heterozygous and homozygous carriers, respectively, have been identified in East Asians (who have a lower CYP3A activity) (Elens et al., 2011; Wang et al., 2011; Okubo et al., 2013). Two alleles associated with no active enzyme, *CYP3A4^∗^20* and *CYP3A4^∗^26*, have also been identified (Westlind-Johnsson et al., 2006; Werk et al., 2014). Recent screening of over 1000 Han Chinese for mutations in CYP3A4 found seven novel exonic variants (*CYP3A4^∗^28-^∗^34*) (Hu et al., 2017).

Midazolam clearance or an erythromycin breath test may be used *in vivo* to measure the activity of CYP3A enzyme in both the intestinal epithelium and liver (Goh et al., 2002). Alfentanil is demethylated by CYP3A4 and may be a useful CYP3A probe due to the pupillary response to alfentanil (Baririan et al., 2005; Klees et al., 2005). Other probes for *in vivo* CYP3A activity include: alprazolam (4-hydroxylation), cortisol (6-β hydroxylation), dextromethorphan (*N*-demethylation), diazepam (*N*-demethylation), nifedipine (oxidation), terfenadine (C-hydroxylation), testosterone (6-β hydroxylation), and triazolam (1-hydroxylation) (Jurica and Sulcova, 2012). Itraconazole and ketoconazole are potent CYP3A4 inhibitors (Jurica and Sulcova, 2012). Due to the presence of multiple substrate binding domains within CYP3A4, the use of at least two structurally unrelated probe substrates is recommended when investigating inhibition effects; crystal structures show that multiple substrate/inhibitor molecules may be simultaneously bound (Korzekwa et al., 1998; Schrag and Wienkers, 2001; Tucker et al., 2001; Ekroos and Sjogren, 2006; Foti et al., 2010).

Although various functional genetic variants have been identified as above outlined, these do not account for the degree of phenotypic variation in enzyme activity seen at the population level. The major mechanisms for the regulation of CYP3A expression in fact appear to be epigenetic, including DNA methylation (Dannenberg and Edenberg, 2006), histone acetylation, and miRNA-mediated mechanisms. In the 5’-region of CYP3A4 gene, histone acetylation occurs in response to the pregnane X receptor (PXR) agonist rifampicin (Xie et al., 2009). CYP3A4 is also regulated in the promoter region of the constitutive androstane receptor (CAR) in response to dexamethasone at a lower rate of expression (Assenat et al., 2004). In addition, hepatocyte nuclear factor 4α can regulate the gene expression of PXR and CAR mediated xenobiotic induction of CYP3A4 (Tirona et al., 2003). In regard to miRNA-mediated mechanisms, miR-27b regulates CYP3A4 expression by binding to the 3’untranslated region (UTR) of CYP3A4 mRNA (Pan et al., 2009), miR-148a regulates other liver specific genes by binding to the 3’UTR of PXR mRNA (Takagi et al., 2008), and the vitamin D receptor (VDR, also an indirect modulator of CYP3A) may be downregulated by miR-27b (Li et al., 2015). Targets genes of the PXR are *CYP3A4*, *CYP2B6*, *MDR1*, members of UGT superfamily, multidrug resistance-related protein-3 (MRP3), and organic anion transporting polypeptide-2 (OARP-2) transporters (Klaassen and Slitt, 2005; Tolson and Wang, 2010) in multiple cell types. P-glycoprotein expression at the blood brain barrier is regulated by PXR activation (Bauer et al., 2004). The PXR is also known as the steroid and xenobiotic receptor (SXR); tamoxifen activates both *CYP3A4* and *MDR1* gene expression through the PXR/SXR in the breast cancer cells (Nagaoka et al., 2006). CAR, PXR, and VDR are members of the nuclear receptor family that also includes FXR, LXR, RXR, and PPARα, which together participate in the complex coordinated regulation of transcription of drug metabolizing enzyme and transporter genes (Czekaj, 2000; Czekaj and Skowronek, 2012). Genetic variants in nuclear receptors contribute to interindividual differences in response to drugs that are metabolized by CYP3A enzymes (Lamba and Schuetz, 2008).

The *CYP3A5^∗^3* (6981A>G) and *CYP3A5^∗^6* (14685G>A) splice site variants are associated with no functional protein (Kuehl et al., 2001; PharmVar CYP3A5 Page, retrieved from https://www.pharmvar.org/gene/CYP3A5). The *CYP3A5^∗^7* variant (27126_27127insT) is also associated with CYP3A5 poor metabolizer status (Hustert et al., 2001).^5^ The majority (80–85%) of White people are *CYP3A5^∗^3/^∗^3* genotype, which means they are CYP3A5 poor metabolizers (van Schaik et al., 2002). Owing to this and other factors affecting CYP3A expression (see below), CYP3A5 is expressed more frequently in those of African descent compared to Whites (55% vs 33% in one study of 47 livers Kuehl et al., 2001), However, as the lists of medications metabolized by CYP3A4 and by CYP3A5 overlap with each other and the sum of the activity in both of these enzymes is the total CYP3A activity, for many medications CYP3A4 is able to substitute for CYP3A5 in those who are CYP3A5 poor metabolizers. For those who are CYP3A5 extensive (normal) metabolizers, they require lower than the usual dose (of relevant medications such as tacrolimus) prescribed for Whites (Birdwell et al., 2015). Functional effects of combined CYP3A4 and CYP3A5 enzyme deficiency may be marked (Werk et al., 2014).

Whilst *CYP3A7* is mainly found in embryonic, fetal, and newborn liver, it may persist; it metabolizes dehydroepiandrosterone and its sulfate (DHEA-S). Persistent CYP3A7 expression in adults and lower levels of DHEA-S in women with polycystic ovary syndrome has been associated with a promoter variant, *CYP3A7^∗^1C* (Goodarzi et al., 2008). Two pseudogenes are found between *CYP3A7* and *CYP3A5* (*CYP3A7-3AP1* and *CYP3A7-CYP3AP;* Nelson et al., 2004; CYP3A5 RefSeqGene on chromosome 7, 2020, retrieved from https://www.ncbi.nlm.nih.gov/nuccore/NG_007938.2).

Of the total CYP3A hepatic content, *CYP3A43* represents a relatively low proportion. Variants in this gene have nonetheless been identified and analyzed for association with clearance of antipsychotics (Variant Annotations). A frameshift mutation is present (c.74delA from the sequence start or c.-30delA from the ATG start, rs61469810), leading to a premature stop codon, a missense mutation (c.1018C>G/P340A, rs680055), and other silent/non-functional mutations. Increased olanzapine clearance in association with rs472660 AA genotype in the CATIE sample was found in an analysis of *CYP3A43* markers available on a particular array (the Affymetrix 500K) (Bigos et al., 2011). The A variant appears more frequent in those of African descent; after accounting for *CYP3A43* genotype, race was no longer a significant predictor of olanzapine clearance.

## Flavin-Containing Monooxygenase

There are six human FMOs (Krueger et al., 2002), encoding enzymes FMO1-5 (the sixth gene is a pseudogene). FMO substrates include CPZ, trifluoperazine, prochlorperazine, promazine, promethazine, and other phenothiazines (Lomri et al., 1993), amphetamines, clomipramine, clozapine, desipramine, imipramine, ketoconazole, methamphetamine, moclobemide, olanzapine, ranitidine, and tamoxifen (Beedham, 1997; Motika et al., 2007; Foti and Dalvie, 2016).

*FMO1* is expressed in adult kidney, intestine, and fetal liver (Yeung et al., 2000). Lower quantities are found in other organs such as the ovaries, testis, adrenal gland and bladder. Substrates include psychotropics mentioned above, disulfiram, nicotine, and pesticides (Phillips and Shephard, 2017). Some of the variability in *FMO1* expression can be accounted for by a promoter SNP (characterizing the *FMO1^∗^6* allele), which has a frequency of 30, 13, and 11% in Hispanics, those of African descent, and Europeans, respectively.

### Flavin-Containing Monooxygenase 2

Flavin-containing monooxygenase 2 (*FMO2*) is expressed in the lungs. The majority of Whites and Asians are homozygous for a non-functional allele: *FMO2*^∗^2A (a C>T mutation at position 1414 that results in a premature stop codon). The wild-type (*FMO2^∗^1*) haplotype is found in African-Americans (26%), Puerto Ricans (7%) and Mexicans (2%) (Whetstine et al., 2000; Furnes et al., 2003). In some populations in Africa, the frequency approaches 50% (Veeramah et al., 2008). The functional haplotype protects against toxicity caused by organophosphate insecticides, however, it also increases the risk of pulmonary toxicity for chemicals containing thioureas. It can metabolize drugs including nicotine, prochlorperazine, and trifluoperazine (Krueger and Williams, 2005) and is responsible for activating anti-tubercular drugs. Hormones including gonadal hormones (and possibly corticosteroids – a glucocorticoid responsive element has been found in the 5’flanking region of the rabbit *FMO2* gene) regulate *FMO2* expression.

### Flavin-Containing Monooxygenase 3

Flavin-containing monooxygenase 3 (FMO3)is present mainly in the liver; lower concentrations can be found in the lungs, kidneys, small intestine, and brain (Chen et al., 2016). Substrates include amphetamine, chlorpromazine, clozapine, imipramine, methamphetamine, and nicotine. Interindividual and interethnic protein concentration variability can be partially explained by the multiple SNPs that have been identified in the *FMO3* gene (Cashman and Zhang, 2002; Krueger et al., 2002). These lead to amino acid substitutions or absence of functional protein, and are associated with the autosomal recessive hereditary condition of trimethylaminuria and milder forms thereof (Mackay et al., 2011). One such variant (Glu158Lys or E158K) may be associated with mild trimethylaminuria and potentially greater neurotoxicity of amphetamine and methamphetamine (which are metabolized to a greater extent to hydroxylamine metabolites by the E158K compared to the wild-type enzyme) (Motika et al., 2007). Trimethylaminuria may be associated with various neuropsychiatric presentations, ranging from depression, anxiety, suicidality, paranoia, addiction (Ayesh et al., 1993) to seizures (McConnell et al., 1997). Flavin-containing monooxygenase 3 converts trimethylamine to trimethylamine *N*-oxide, which is excreted in the urine, but also appears in the sweat, saliva, breath, and vaginal secretions.

Flavin-containing monooxygenase 3 activity is affected by hormones (the symptoms of trimethylaminuria can be worse in women, especially after puberty, after taking oral contraceptives, and at the time of the menstrual cycle or perimenopause), dietary content (choline, lecithin, tyramine), and intestinal bacterial overgrowth (reducing trimethylamine *N*-oxide to trimethylamine). Brussel sprout consumption acts as an inhibitor of FMO3, decreasing FMO3 activity, and can worsen the trimethylaminuria condition (Motika et al., 2007). For individuals deficient in FMO3, supplementation with folate and riboflavin is indicated (Motika et al., 2007). Choline and lecithin are found in egg yolk, kidney, liver, legumes, peas, salt-water fish, shellfish, and soybeans. The enzyme is subject to competitive inhibition effects (e.g., by CPZ, and imipramine) (Adali et al., 1998). Methimazole is a potent inhibitor of both FMO1 and FMO3. Recent publications have shown that *N*-oxidation of nicotine mediated by FMO1 and FMO3 occurs in the brain, and, moreover, that functional variation in FMO3 (rs2266780, E308G) is associated with nicotine dependence (Teitelbaum et al., 2018).

## Esterases and Microsomal Epoxide Hydrolases

The metabolism of approximately 10% of therapeutic drugs with ester, amide, and thioester functional groups is catalyzed by esterases (Fukami and Yokoi, 2012). A common family of esterases, the B-esterase family, includes cholinesterases such as acetylcholinesterase (AChE). Cholinergic transmissions are regulated by AChE, selectively inactivating acetylcholine released from the presynaptic cleft of neurons of the brain, skeletal muscle, and the autonomic nervous system (Hasin et al., 2005).

Epoxide hydrolases are a family of enzymes that transform reactive epoxide molecules into more stable and more soluble diols (El-Sherbeni and El-Kadi, 2014). *EPHX1* encodes mEH. It is a highly polymorphic gene, with over 100 SNPs identified. Enzyme activity is reduced by 40% in the variant with the c.337 T>C SNP, and 25% in the c.416 A>G variant (Caruso et al., 2014). Alcohol dependence has been associated with these SNPs (Bhaskar et al., 2013). Possibly altered response to carbamazepine and warfarin has been associated with genetic variants in *EPHX1* (Nakajima et al., 2005; Puranik et al., 2013; Caruso et al., 2014; Daci et al., 2015; Liu et al., 2015).

## Phase II Metabolism

**FIGURE 1:**
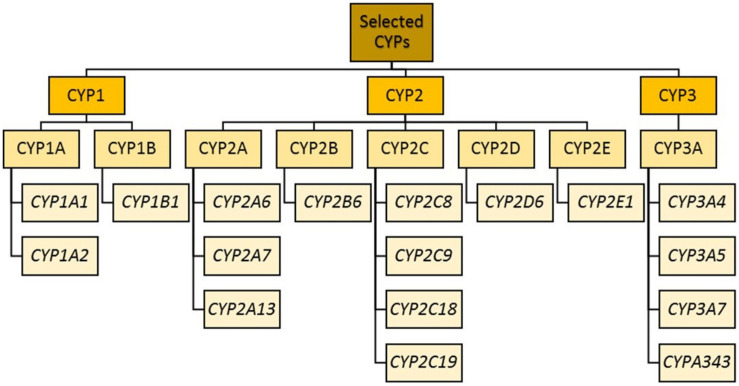
CYP enzyme families most relevant to psychotropic drug metabolism.

Phase II enzymes include EH, glutathione S-transferases (GSTs), *N*-acetyltransferases, sulfotransferases, and UDP-glucuronosyltransferases (UGTs) (Jancova et al., 2010), the actions of which lead to the formation of more hydrophilic molecules for renal or biliary excretion (or further metabolite activation, which may be associated with toxicity). Within these groups, the enzymes most relevant to psychotropic drug metabolism are shown in Figure 1.

Enzymes in this phase can be classified as either type I or type II conjugation. In type I, an activated conjugating agent combines with the substrate to yield a conjugated product through the addition of functional motifs (such as acetate, glutathione, glucuronate, or sulfate), consequently increasing the xenobiotic polarity and hydrophilicity. In type II conjugation, the substrate is activated and then combined with a moiety such as a methyl group or amino acid (Jančová and Šiller, 2012).

### Type I Conjugation

Cytosolic enzymes expressed in the liver and intestine are encoded by the genes NAT1 and NAT2. NAT1 shows additional wide tissue distribution (Windmill et al., 2000; Sim et al., 2007, 2014) and is expressed in fetal and neonatal tissue, while *NAT2* is not expressed until approximately a year later (Pacifici et al., 1986; Pariente-Khayat et al., 1991). The substrate specificity of NAT1 and NAT2 overlaps. Moreover, genetic variants in one are linked to those in another; they can therefore act in a concerted fashion to “cox and box” against evolutionary selection pressures with mutually compensatory mechanisms.

Glutathione S-transferases are relevant not only to drug metabolism, but also to detoxification of reactive intermediates such as those formed by catecholamine peroxidation (aminochrome, dopachrome, adrenochrome) in the defense against oxidative stress (Jančová and Šiller, 2012). Glucuronosyltransferases are located mainly on the luminal membrane of the endoplasmic reticulum, and act in concert with the CYP enzymes present on the cytosolic surface (Ouzzine et al., 1999; Ishii et al., 2007). Some glucuronidated products are less active; others, such as morphine-6-glucuronide, are pharmacologically active (Gong et al., 1991). UDP-glucuronosyltransferase substrates of relevance to neuropsychiatry include: apomorphine, dopamine, ethanol, lamotrigine, morphine, oxazepam, serotonin, and valproic acid (de Leon, 2003; Ouzzine et al., 2014). In the; brain, UGTs are found in the endothelial cells and astrocytes of the blood–brain barrier, as well as in the pituitary, pineal, neuro-olfactory tissue, and circumventricular organ (Ouzzine et al., 2014).

UDP-glucuronosyltransferase nomenclature is similar to that of the CYPs, with the UGT1 and UGT2 subfamilies being the most relevant for drug metabolism (Mackenzie et al., 2005).^6^ UDP-glucuronosyltransferase activity is influenced by factors including cigarette smoking, obesity, age, and gender (Liston et al., 2001). Although relevant to the metabolism of both endogenous and exogenous substances, there are to date relatively few studies that have both therapeutic drug monitoring data and UGT enzyme phenotype (Stingl et al., 2014). UGT1A4, UGT1A6, and UGT2B7 are relevant to the clearance of multiple psychotropics including valproic acid, lamotrigine, olanzapine, clozapine, paliperidone, chlorpromazine, and loxapine (Stingl et al., 2014; Mazerska et al., 2016). These enzymes are also expressed in the brain (King et al., 1999; Ghosh et al., 2013). Elevated plasma lamotrigine has been observed when given in combination with valproic acid, which has been attributed to competitive inhibition of UGT1A4 and UGT2B7 metabolism (Gidal et al., 2003; Rowland et al., 2006). Reduced lamotrigine clearance is seen in patients with benign unconjugated hyperbilirubinemia (Gilbert’s syndrome), which is associated with the *UGT1A1^∗^28* haplotype in Whites and the *UGT1A1^∗^6* haplotype in Asians (Akaba et al., 1998; Beutler et al., 1998; Barbarino et al., 2014; UGT1A1 and common exons allele nomenclature). Some associations as yet awaiting replication have been found: elevation of valproic acid clearance in carriers of *UGT1A3^∗^5* (Chu et al., 2012), reduction of lamotrigine clearance by 60% in those homozygous for *UGT2B7^∗^2* (Blanca Sánchez et al., 2010), and a doubling of the clearance in *UGT1A4^∗^3* heterozygotes (Gulcebi et al., 2011). A doubling in the direct glucuronidation of olanzapine is seen in those of *UGT1A4^∗^3/^∗^3* genotype, with a reduction in those with at least one *UGT2B10^∗^2* variant (Erickson-Ridout et al., 2011). Individuals homozygous for the *UGT2B15^∗^2* haplotype have 50% lower benzodiazepine clearance (lorazepam, and the diazepam CYP metabolite oxazepam) (Chung et al., 2005; He et al., 2009). The 142T>G *UGT1A4* polymorphism is associated with reduced clozapine and olanzapine concentrations (Mori et al., 2005; Ghotbi et al., 2010).

The human sulfotransferase superfamily of enzymes contains at least 13 members, with partially overlapping substrate specificity and tissue distributions (Riches et al., 2009). Some sulfo-conjugates are active; however, sulfo-conjugation usually reduces biological activity. For example, pregnenolone sulfate blocks the activation of GABAA receptors (Majewska et al., 1988), although it is a positive allosteric modulator of the NMDA receptor (Wu et al., 1991).

### Type II Conjugation

Many exogenous and endogenous compounds can undergo *N*-, *O*-, *S*-, or arsenic-methylation (Feng et al., 2010). The co-factor required is *S*-adenosylmethionine (SAM), formed from ATP and L-methionine. Catechol *O*-methyltransferase (COMT) is a magnesium-dependent enzyme (Axelrod, 1957) that has a key role in the modulation of functions such as cardiovascular function, cognition, and pain processing, which are catechol dependent. Catechol *O*-methyltransferase is involved in the inactivation of catecholamine neurotransmitters (dopamine, noradrenaline), catechol-estrogens and other catechol drugs such as L–DOPA (Weinshilboum et al., 1999). There two forms of COMT: a cytoplasmic soluble form (S-COMT), and a membrane-bound form (MB-COMT), located on the cytosolic side of the endoplasmic reticulum. S-COMT is found in the liver, intestine and kidney (Taskinen et al., 2003), whereas the MB-COMT is more highly expressed in the central nervous system (Tunbridge et al., 2006). The *COMT* Val158Met (rs4680) polymorphism has been associated with a variety of relevant phenotypes including cognition (Goldman et al., 2009), pain tolerance (Goldman, 2014), and age of onset of psychosis after adolescent cannabis consumption (Caspi et al., 2005; Lodhi et al., 2017).

## Phase III Elimination

The final step in drug processing is the export of compounds away from the interior of cells in an energy-dependent manner. Metabolized molecules are transported by the ATP-binding cassette (ABC) superfamily (Hugo Gene Nomeclature Committee, 2020); energy (ATP) is used to transport substances out of the cell against a concentration gradient in multiple different organs including the brain during this phase (Dean et al., 2001; Borst and Elferink, 2002; Doring and Petzinger, 2014).

*ABCB1* (previously called *MDR1*) was the first member to be cloned (Riordan et al., 1985; Roninson et al., 1986; Ueda et al., 1987), with the encoded protein (p-glycoprotein or p-gp) being called multidrug resistance protein owing to the observation that it was overexpressed in tumor cells with resistance to multiple chemotherapeutic agents.

As reviewed by Hodges et al. (2011), this protein has a complex structure. Two homologous halves each contain six transmembrane domains, which surround an aqueous pore within which conserved residues recognize a diverse range of substrates. It can distinguish stereoisomers and bind multiple substrates simultaneously in close proximity to each other, with associated allosteric, competitive and non-competitive inhibition, and cooperativity between substrates. Polymorphisms in *ABCB1* and their role in response to antidepressants have been reviewed (Peters et al., 2009; Hodges et al., 2011).

The most commonly studied variant is a triallelic SNP (c.3435T>C, c.3435T>G and c.3435T>A, reverse strand) (rs1045642). The c.3435T>C (or C3435T) is a synonymous SNP that is in linkage disequilibrium with another synonymous SNP (C1236T, rs1128503) and a coding SNP (G2677T, ClinVar database: rs2032582). Haplotypes such as C1236T-G2677T-C3435T that include the C3435T have been associated with reduced inhibition by cyclosporin and verapamil of p-gp mediated substrate (in this case paclitaxel) efflux, with differences being more pronounced at higher levels of p-gp expression (Kimchi-Sarfaty et al., 2007). Sensitivity to rapamycin inhibition was not altered (Kimchi-Sarfaty et al., 2007). The altered sensitivity appeared to be owing to conformational change (as indicated by the use of a conformation sensitive monoclonal antibody, Kimchi-Sarfaty et al., 2007). The 3435C variant frequency varies between 34 and 90% in different ethnic groups (reviewed in Borst and Elferink, 2002; Hodges et al., 2011).

P-gp substrates include many psychotropic drugs (e.g., fluvoxamine, paroxetine, venlafaxine, amitriptyline, desipramine, trimipramine, doxepin, olanzapine, risperidone (RIS), paliperidone, CPZ, diazepam, lamotrigine, carbamazepine, and phenytoin (Uhr and Grauer, 2003; Gunes et al., 2008; Uhr et al., 2008; Aller et al., 2009; O’Brien et al., 2012; de Klerk et al., 2013; Palleria et al., 2013; Lund et al., 2017; UniProtKB). Data on citalopram vary depending on the model system and fluoxetine and mirtazapine are not p-gp substrates (reviewed in Peters et al., 2009). The list of non psychotropic substrates is extensive (reviewed in Hodges et al., 2011).

Overlapping substrate specificity with other ABC transporters is present. P-gp is expressed on the apical membrane of the intestine from the duodenum to the rectum, being coregulated with CYP3A4 in the duodenum and jejunum, and coregulated with CYP3A5 in the rectum and sigmoid colon (von Richter et al., 2004; Ufer et al., 2008; Cascorbi, 2011; Fromm and Kim, 2011). It shares substrate specificity with CYP3A4, and both are regulated by St John’s Wort (Johne et al., 1999), amongst other drugs. High affinity substrates such as verapamil also inhibit p-gp at the blood–brain barrier, causing drugs such as loperamide to affect the central nervous system (an anti-diarrheal medicine that normally has no central nervous system effects) (Elsinga et al., 2004). A review on the topic of p-gp and its relevance to drug–drug interactions (DDI) underlines that data observed *in vitro* may not always be reflected by that seen in clinical practice *in vivo* (Lund et al., 2017). *In vivo* data indicate that carbamazepine and phenytoin are p-gp inducers, while fluvoxamine and paroxetine are p-gp inhibitors (Lund et al., 2017).

In the liver, p-gp levels vary 50-fold. More than 51000 mutations in the *ABCB1* gene region including over 137 missense^7^ variants have been identified. Pharmacogenetic studies to date have often focused on a limited number of SNPs, such as the three described above. Data up to 2009 in regard to associations with response to antidepressants were summarized as equivocal (Peters et al., 2009), with a subsequent pharmacogenetically guided clinical trial (Singh, 2015) and a meta-analysis including this trial concluding in favor of this gene potentially having a role in pharmacogenetically guided treatment (Bousman et al., 2019b). Singh (2015) suggests that *ABCB1* should be considered together with *ABCC1*. Fabbri and Serretti (2015); Lett et al. (2016), and Amare et al. (2017) include data on *ABCB1* in their antidepressant response reviews, with a recent study in an E. Asian population reporting an association with response to serotonin noradrenaline reuptake inhibitors (SNRIs; Shan et al., 2019). In a review on clozapine, Krivoy et al. (2016), concluded that *ABCB1* genotypes including the C3435T were associated with clozapine concentration and response.

While many studies have focused on the above outlined SNPs, particularly the C3435T, an approach in which haplotypes are linked to transporter phenotypes and systematically cataloged to inform clinical association analyses is surely desirable. For example, using *in silico* molecular techniques to predict amino acid residues that bind to psychotropics and hence which mutations might be investigated for clinical association analyses could be an informative approach. Further, elucidating mechanisms by which different co-administered medications might interact at p-gp would be helpful.

## Pharmacogenetic Associations Relevant to Psychiatry

After initial prescription, psychiatric medicines have a 40–60% failure rate (Correll et al., 2015). Implementation of pharmacogenetics can improve current methods of physician judgment and therapeutic trials. Challenges to data standardization are prevalent (de Leon, 2009; Malhotra et al., 2012; Altman et al., 2013; Bousman and Hopwood, 2016; Bousman et al., 2018). To address this, the Clinical Pharmacogenetics Implementation Consortium (CPIC) was created in 2009 by PharmGKB and the Pharmacogenomics Research Network (Relling and Klein, 2011) to provide prescribing guidelines for genetic variants. CPIC consists of four levels of recommendation concerning drug-gene pairs (Caudle et al., 2016).^8^ Recommendation levels are denoted based on literature reviews presented to the CPIC writing committee. Evidence classifications include “high,” “moderate,” or “weak,” based on design, quality, and generalizability of the research. Therapeutic recommendations are graded as “strong,” “moderate,” or “optional” (Caudle et al., 2014). Guidelines focus on gene–drug pairs where the prescribing recommendations are actionable (level A or B) (Table 1).^9^

**TABLE 1:** Mental health medications: Clinical Pharmacogenetics Implementation Consortium (CPIC) evidence levels, pharmacogenomic FDA label, and associated genes.

Drug	CPIC level	PharmGKB level of evidence	PGx on FDA label	Gene
Amitriptyline	A	1A	Actionable PGx	*CYP2D6*
	A	1A	–	*CYP2C19*
Aripiprazole	B	3	Actionable PGx	*CYP2D6*
Atomoxetine	A	1A	Actionable PGx	*CYP2D6*
Brexpiprazole	B	–	Actionable PGx	*CYP2D6*
Carbamazepine	A	1A	Genetic testing required	HLA-B*1502
	A	1A	Actionable PGx	HLA-A*3101
Citalopram & Escitalopram	A	1A	Actionable PGx	*CYP2C19*
Clomipramine	B	1A	Actionable PGx	*CYP2D6*
	B	2A		*CYP2C19*
Desipramine	B	1A	Actionable PGx	*CYP2D6*
Doxepin	B	1A	Actionable PGx	*CYP2D6*
	B	3	Actionable PGx	*CYP2C19*
Fluvoxamine	A	1A	Actionable PGx	*CYP2D6*
Imipramine	B	1A	Actionable PGx	*CYP2D6*
	B	2A	–	*CYP2C19*
Nortriptyline	A	1A	Actionable PGx	*CYP2D6*
Paroxetine	A	1A	Informative PGx	*CYP2D6*
Perphenazine	B/C	–	Actionable PGx	*CYP2D6*
Pimozide	B	4	Genetic testing required	*CYP2D6*
Protriptyline	B	–	Actionable PGx	*CYP2D6*
Trimipramine	B	1A	Actionable PGx	*CYP2D6*
	B	2A	–	*CYP2C19*
Valproic acid	B	3	Genetic testing required	*POLG*
Venlafaxine	B	2A	Actionable PGx	*CYP2D6*
Vortioxetine	B	3	Actionable PGx	*CYP2D6*

*–Denotes information unavailable or undecided to date on CPIC guidelines.*

Prior to the implementation of CPIC, in 2005, the Royal Dutch Pharmacists Association established a similar body, the Dutch Pharmacogenetics Working Group (DPWG), to provide prescribing guidelines for specific gene–drug pairs to physicians and pharmacists in the Netherlands and now used internationally.^10^ Similar to CPIC, evidence for strength of a prescribing recommendation (such as to avoid a particular drug in the presence of a specific genotype) is ranked on a 0–4 scale (Bank et al., 2018). While there is significant overlap between the recommendations offered by these two organizations, some differences in therapeutic recommendations can be found (Bank et al., 2018; van Westrhenen et al., 2020).

Below are provided further details for pharmacogenetic associations for specific classes of medications relevant to psychiatry.

### Mood Stabilizers

There is significant interindividual variation in treatment response and adverse reactions to mood stabilizers (Murru et al., 2015; Tang and Pinsky, 2015; Pisanu et al., 2016). The current CPIC gene–drug pair list includes carbamazepine, oxcarbazepine and valproic acid (Saruwatari et al., 2010; Drozda et al., 2014), with guidelines available for the first two (Relling et al., 2011; Phillips et al., 2018).

Carbamazepine and oxcarbazepine are anticonvulsants approved for treating epilepsy, trigeminal neuralgia, and bipolar disorder (Phillips et al., 2018). Therapeutic drug monitoring for anticonvulsants is well-established. Both share dose-dependent (type A) ADRs including ataxia. Type B ADRs (not predictable from the pharmacology) are potentially lethal and include osteoporosis, aplastic anemia, and Stevens-Johnson syndrome/toxic epidermal necrolysis (SJS/TEN).

Genetic variants having actionable levels with carbamazepine and oxcarbazepine are *HLA-B^∗^15:02, HLA-A^∗^31:01* and *SCN1A* (Relling and Klein, 2011; Phillips et al., 2018). Associations have been shown in Asians with *HLA-A^∗^31:01* and carbamazepine induced SJS/TEN (Ferrell and McLeod, 2008; Stern and Divito, 2017). A 2004 report in Han Chinese found that the SJS/TEN frequency reduced to 0% after *HLA-B^∗^1502* genotype pre-screening (Chung et al., 2004). East Asians exhibit the highest *HLA-B^∗^15:02* haplotype frequency (∼15%) compared to other populations (>1%). In Hong Kong, Taiwan, and Thailand, testing for this haplotype prior to prescribing carbamazepine and oxcarbazepine is standard practice (Chen et al., 2014; Sukasem and Chantratita, 2016; Lin et al., 2018). However, recent data indicate that the *HLA-B^∗^15:02* frequency in other populations may also be high enough to justify testing in other ethnic groups (Fang et al., 2019). *HLA-A^∗^31:01* haplotype frequency also varies by ethnicity, being up to 15% in most Asian and White groups and infrequent in those of African descent (Fan et al., 2017).

Valproic acid (or its derivative, divalproex sodium, which is converted to valproic acid in the stomach) increases the levels of γ-aminobutyric acid (GABA) in the brain, blocking voltage gated ion channels (particularly calcium and sodium), and inhibiting histone deacetylase enzymes, including HDAC1. Genetic factors are associated with differential efficacy and ADRs (Kasperaviciūte and Sisodiya, 2009; Löscher et al., 2009; Fricke-Galindo et al., 2018). Hepatic metabolism occurs via CYP-mediated oxidation, glucuronidation, and mitochondrial oxidation (Johannessen and Landmark, 2010; Chatzistefanidis et al., 2012; Ghodke-Puranik et al., 2013).

ADRs associated with valproic acid include hepatotoxicity, mitochondrial toxicity, and potentially fatal hyperammonemia encephalopathy, among others (Linnet and Wiborg, 1996; Johannessen and Landmark, 2010; Singh et al., 2015). Valproic acid is contraindicated in patients with disorders secondary to mutations in DNA polymerase gamma (*POLG*), which replicates mitochondrial DNA. Patients with POLG-related disorders have elevated risk of fatal hyperammonemia encephalopathy. The onset of such may vary from childhood to late adulthood. It is therefore contraindicated in children with clinical suspicion of a hereditary mitochondrial disorder. In those over two years of age with suggestive symptoms (such as migraine with defined types of aura), valproate *POLG* testing is required,^11^ and it should be used if the testing is negative, other anticonvulsants have failed, and liver function is monitored.

### Antipsychotics

In this section, pharmacogenetic data available for some specific medications are used to illustrate key applicable principles.

#### Perphenazine

Perphenazine undergoes substantial first-pass hepatic phase I and II metabolism. Serum concentrations vary widely due to polymorphisms in multiple phase I enzymes: up to 30-fold in CYP2D6 NMs (Linnet and Wiborg, 1996). Initial studies showed that after 4–5 weeks, improvement was associated with plasma perphenazine concentrations above 2 nmol/l, while extrapyramidal effects occurred at concentrations above 3 nmol/l (Hansen, 1981; Hansen et al., 1982; Hansen and Larsen, 1983). In a larger study of over 200 patients, a wider therapeutic range (2–6 nmol/l) was suggested (Hansen and Larsen, 1985). Perphenazine binds dopamine D_2_ and alpha-_1_/alpha-_2_ receptors with 70 and 50% antagonism. The main active metabolite, 7-hydroxyperphenazine, binds dopamine D2 and alpha-1/alpha-2 receptors with 70 and 50% the antagonism of perphenazine (Hals et al., 1986). It is formed in a reaction catalyzed by CYP2D6, with other metabolites including *N*-dealkylated perphenazine (formed in part by other CYPs), and perphenazine sulfoxide (Dahl-Puustinen et al., 1989; Olesen and Linnet, 2000). Compared to perphenazine, the concentration of perphenazine sulfoxide is in the same range, while *N*-dealkylated perphenazine is approximately three times that of perphenazine (Hansen et al., 1979). At therapeutically relevant concentrations of perphenazine, CYP3A4 accounts for about 40% of the *N*-dealkylation, with CYP isoforms 1A2, 2C19 and 2D6 contributing 20–25% (Olesen and Linnet, 2000).

The peak serum concentration and the AUC of perphenazine for CYP2D6 PMs is about 3 and 4 times, respectively that of NMs in single dose kinetics (Dahl-Puustinen et al., 1989), and at steady state, the median concentration-to-dose ratio of perphenazine in CYP2D6 PMs is about twice that of NMs, with patients on concomitant inhibitors showing a median concentration in between the two groups (Linnet and Wiborg, 1996). Jerling et al. (1996) conducted a study of patients during treatment; CYP2D6 genotype was shown to significantly predict the oral clearance of perphenazine (patients with two *CYP2D6* PM alleles having lower clearance than heterozygote PMs or NMs) (Jerling et al., 1996).

It would be expected that individuals deficient in CYP2D6 or on potent CYP2D6 inhibitors, higher perphenazine concentrations would be found and hence more adverse effects, whilst in CYP2D6 UMs, there would be lower concentrations, with less adverse effects and potentially a lower therapeutic efficacy. Consistent with this, paroxetine, a potent CYP2D6 inhibitor (Lam et al., 2002), increases the AUC of perphenazine 7-fold in NMs, which is associated with increased side effects (Ozdemir et al., 1997).

#### Pimozide

Since 1984 pimozide has been used to treat Gilles de la Tourette’s syndrome (Pringsheim and Marras, 2009), and also to treat psychotic disorders. Its use has been limited owing to an ADR of prolongation of the QT interval on the electrocardiogram, which is associated with risk for Torsades de Pointes (a type of ventricular fibrillation that may cause sudden cardiac death) (Fulop et al., 1987; Committee on Safety of Medicines-Medicines Control Agency, 1995). In an isolated rabbit heart, this effect was shown to be attributable to pimozide itself, not to metabolites (Flockhart et al., 2000); this is due to an effect of the drug on potassium channels encoded by the human ether-a-go-go-related gene (*HERG*, otherwise known as *KCNH2*), which is responsible for the delayed repolarization current in the heart.

It is important to determine which cytochromes might contribute to the pimozide concentration profile. *In vitro* analyses showed that the formation of the major metabolite, 1,3-dihydro-1-(4-piperidinyl)-2H-benzimidazol-2-one (DHPBI), by *N*-dealkylation was primarily dependent on CYP3A4, with a lesser contribution by CYP1A2 (Desta et al., 1998). CYP2D6 may also play a role, but due to it being inhibited by pimozide, it was not possible to draw a conclusion regarding this from this *in vitro* study.

Case reports of interactions between pimozide and CYP2D6 inhibitors such as paroxetine and fluoxetine (Ahmed et al., 1993; Horrigan and Barnhill, 1994), as well as investigation of differential interaction with clarithromycin (an inhibitor of CYP3A) by CYP2D6 status led to recognition that CYP2D6 was a major contributor to the *in vivo* pharmacokinetics of pimozide (Desta et al., 1998). The effect of a single dose (6 mg) on the QTc interval (QT interval corrected for heart rate) was measured over time, and showed the greatest increase within the first 20 hours, with NMs showing a larger increase (by nearly 20 ms), followed by a reduction from 20 to 50 h, and then an increase at approximately 60–100 h. The late elevation was more significant in CYP2D6 PMs, women, and clarithromycin-treated individuals, and appeared more sustained than the early increase. Owing to the more sustained nature, the late onset elevation may be more relevant to significant QTc prolongation; the early peak in NMs warrants further investigation in UMs. In CYP2D6 PMs, half-life increased from 29 ± 18 h to 36 ± 19 h, while in NMs, the corresponding values were 17 ± 7 and 23 ± 10 h. For subjects with relevant data, the pimozide induced QTc interval changes coincided with the concentration-time course of pimozide. The prescription of CYP3A inhibitors, such as valproate, is now contraindicated with pimozide. In the above study, interestingly, pimozide rapidly increased plasma prolactin concentration, the maximum increase occurring 4 hours post dose, with a sharp reduction thereafter.

Simulated steady-state pharmacokinetic profiling of pimozide in CYP2D6 PMs, IMs, and NMs led to specification in the FDA label in 2011 that CYP2D6 PMs should not be prescribed more than 4 mg, with the maximum recommended dose in CYP2D6 NMs being 10 mg (Rogers et al., 2012). In the simulated data, 4 mg/day in CYP2D6 PMs was the maximum dose that did not result in plasma concentrations in excess of those observed in CYP2D6 NMs receiving 10 mg/day (Desta et al., 1998). Pimozide is commenced at 0.05 mg/kg (Preskorn, 2012), once daily. If the patient is a CYP2D6 NM and is not on a CYP2D6 inhibitor, the dose may be increased every third day to a maximum of 0.2 mg/kg/day, to a maximum of 10 mg/day. If the CYP2D6 status is not known, *CYP2D6* genotyping should be done before deciding to increase the dose above 0.05 mg/kg/d, which is the maximum dose for a CYP2D6 PM, or if on a CYP2D6 inhibitor such as paroxetine, fluoxetine, and bupropion. Paroxetine will convert 60% of CYP2D6 NMs to PMs at 20 mg daily, while at 40 mg daily, 95% will be phenocopied to PMs (Preskorn, 2003). Phenoconversion (the conversion of an individual’s genetically defined metabolizer status to another status owing to the effect of a pharmacologically active substance) to CYP2D6 PM status by the action of an enzyme inhibitor has been estimated as being 6 times more common than genetically determined CYP2D6 PM status (Preskorn, 2012, 2013).

First pass metabolism of pimozide includes both the gut and the liver as CYP3A represents 70% and 30% of the total CYP450 in the intestine and the liver, respectively (Kolars et al., 1994; Shimada et al., 1994). Metabolism will be subject to the influence of gut microbiota, diet, and other factors including hormones (CYP3A4 being subject to regulation by the PXR and CAR) (Lamba et al., 2005; Pan et al., 2009).

The drug label does not currently include dosing recommendations for CYP2D6 UMs; further research including genotyping CYP2D6 is required for pimozide, and other CYP2D6 metabolized medications.

It is suggested that *CYP3A4* also be genotyped for pimozide treatment, given its association with sudden cardiac death. It has a less clear genotype–phenotype relationship (with no updated data on PharmVar), and thus has not yet been introduced into clinical guidelines. In the absence of genotyping, probe drugs such as nifedipine may be utilized to test the activity of multiple CYPs (de Andrés et al., 2014); however, such estimation of CYP3A4 phenotype is influenced by any concomitant medication and/or dietary effects.

#### Haloperidol

Haloperidol (HAL) is a butyrophenone and first-generation antipsychotic (FGA) drug that acts as a dopaminergic antagonist in the mesolimbic system. It is used to treat a variety of psychiatric conditions, including psychoses (e.g., schizophrenia, schizoaffective disorder, bipolar disorder with mania or psychotic symptoms, substance-induced psychotic disorder) and other conditions with hallucinations (e.g., alcohol withdrawal, delirium, Lewy body dementia). Adverse effects may include tardive dyskinesia, neuroleptic malignant syndrome, and a prolonged QT_*c*_ interval. Two major routes of metabolism, *N*-glucuronidation and *O*-glucuronidation, are effected by UGT enzymes, specifically the former by UGT1A4, and the latter by UGT1A4, UGT1A9, and UGT2B7 (Kato et al., 2012). Various CYP isoenzymes contribute to the metabolic pathways of this medication, most notably CYP3A4, and, to a lesser extent, CYP2D6. Cytosolic carbonyl reductase catalyzes the formation of reduced HAL, which retains 10–20% of the activity of the parent compound. Reduced HAL can be further metabolized by CYP3A4 to a tetrahydropyridine. The reduced drug can also be back-oxidized by CYP3A4 to HAL (Pan et al., 1998; Kudo and Ishizaki, 1999; Tateishi et al., 2000; discussed in Aitchison et al., 1999). Owing to its lipophilicity, HAL is extensively metabolized in humans, with large interindividual variations in pharmacokinetics arising. With a proposed therapeutic range of 5.6–16.9 μg/l in serum (Ulrich et al., 1998), being able to appreciably predict pharmacokinetic parameters in individuals is of utmost importance to optimize efficacy and safety. At lower doses, CYP2D6 contributes to HAL metabolism significantly, but with higher doses, and longer term treatments, CYP3A4 back-oxidation and *N*-dealkylation considerably outweigh the contributions of CYP2D6 (Fang et al., 1997; Pan et al., 1998; Zhou et al., 2009). Nyberg et al. (1995) showed that CYP2D6 PMs exhibited higher plasma concentrations of HAL over a 4-week treatment period with HAL decanoate, as compared to seven NMs in the study. However, Ohnuma et al. (2003) showed that, in a large number of Japanese patients, the presence of neither an enzyme activity-reducing mutation (*CYP2D6^∗^10A*) nor activity-increasing mutations (duplications) in *CYP2D6* alone could appreciably predict HAL concentrations.

Haloperidol is a medication that is CPIC level B (for CYP2D6),^12^ with a guideline currently in progressguidelines. Further, in the DPWG guidelines, there is a recommendation for initial dose to be reduced to 50% in PMs or for selection of an alternative medication based on a metabolic pathway different than CYP2D6. Possible dose adjustments are also mentioned for UMs.^13^ In a study of 70 patients in which the most commonly prescribed medication was HAL, the risk of tardive dyskinesia increased with increasing number of functional *CYP2D6* genes, with UMs showing the highest risk (Koola et al., 2014).

Enzyme induction effects are also relevant for HAL metabolism. First, there is the effect of smoking. From a relevant review (Desai et al., 2001), it may be deduced that smoking increases the clearance of oral HAL (via effects including on CYP1A2), especially at doses lower than 0.5 mg/kg/day. Carbamazepine (which induces several CYPs including the CYP3As) reduces plasma HAL concentration (Hesslinger et al., 1999).

#### Chlorpromazine

Chlorpromazine is a phenothiazine that was the first antipsychotic to be introduced (reviewed in Basu et al., 2007). Its biotransformation includes hydroxylation (by CYP2D6 and CYP1A2), *N*-methylation, *N-N*-didemethylation, *N*-oxidation, *S*-oxidation, and glutathione conjugation. The hydroxylated metabolite can undergo further oxidation leading to formation of an electrophilic quinone imine intermediate, which is capable of mediating toxic effects (by reacting with cellular proteins and DNA) or underdoing glutathione conjugation (Wen and Zhou, 2009). Muralidharan et al. (1996) confirmed the contribution of CYP2D6 to the hydroxylation pathway using quinidine, whilst also showing that *CYP2D6* genetic polymorphism was not the major contributor to inter-individual variability in plasma concentrations. The latter finding was confirmed by Yoshii and colleagues (Yoshii et al., 2000), whose microsomal inhibition studies of chlorpromazine 7-hydroxylation showed that CYP1A2 may play a more important role in the hydroxylation reaction for individuals deficient in CYP2D6. Indeed, Gill et al. (1997) reported that an individual with schizophrenia who was homozygous for the *CYP2D6^∗^4* variant (then known as the “B”) and therefore a PM and had been intolerant and non-compliant with multiple medications settled on a very low dose (50 mg) of chlorpromazine (Gill et al., 1997).

#### Zuclopenthixol

Zuclopenthixol is a thioxanthene derivative used to treat schizophrenia, having high affinity for both D_2_ and D_1_ dopamine receptors (Kumar and Strech, 2005). Its metabolic pathways include sulfoxidation, *N*-dealkylation, and glucuronidation (Zhou et al., 2009), with metabolites not known to have antipsychotic activity.

Dahl et al. (1991) showed that clearance of zuclopenthixol was associated with debrisoquine hydroxylation, and further studies confirmed the role of CYP2D6 in zuclopenthixol metabolism (Zhou et al., 2009). Moreover, PMs had a 1.9-fold higher AUC of zuclopenthixol compared to NMs (*n* = 6 for each group) after a single 6 or 10 mg dose (Dahl et al., 1991). Linnet and Wiborg (1996) found similar results: investigation of phenotypic relationships to zuclopenthixol concentration showed that, in 12 psychiatric patients, CYP2D6 PMs had 60% greater concentrations than NMs, but were similar to NMs who were co-administered CYP2D6 inhibiting drugs.

Furthermore, in another study, psychiatric patients treated with zuclopenthixol who experienced adverse neurological events (tardive dyskinesia, parkinsonism) tended to have a higher frequency of non-functional *CYP2D6^∗^3* and *^∗^4* alleles, but these results did not attain statistical significance (Jaanson et al., 2002).

Zuclopenthixol is CPIC level B,^14^ with a guideline in progress (and also not yet available from DPWG). One review suggests considering dose adjustment (58 and 88% for CYP2D6 PMs and IMs, respectively) or selecting an alternative medication (Stingl et al., 2013).

#### Aripiprazole

Aripiprazole was marketed as the first antipsychotic with dopamine and serotonin partial agonism. In Europe, aripiprazole is indicated for use in the treatment of schizophrenia and treatment of moderate to severe manic and episodes associated with bipolar I disorder and for the prevention of new manic episodes in those whose manic episodes respond to aripiprazole (Koskinen et al., 2010; Abilify 10 mg tablets; Abilify; Summary of Product Information, EMA). Other licensed indications include adjunctive treatment of major depressive disorder, Tourette’s syndrome, and irritability in autism spectrum disorder (Mailman and Murthy, 2010). Global therapeutic efficacy has been measured versus aripiprazole and dehydroaripiprazole serum concentrations, with a reported 68% response rate in those with concentrations of 150–300 ng/ml of aripiprazole, and a 57 and 50% response rate with concentrations less than 150 ng/ml or above 300 ng/ml, respectively (Kirschbaum et al., 2008). Therapeutic drug monitoring (TDM) has “recommended” (level 2 evidence) for aripiprazole by the interdisciplinary TDM group of the Arbeitsgemeinschaft für Neuropsychopharmakologie und Pharmakopsychiatrie (AGNP), with a therapeutic target range of 100–350 ng/ml for aripiprazole, or 150–500 for aripiprazole and dehydroaripiprazole (Hiemke et al., 2018).

Aripiprazole undergoes substantial first pass metabolism in the liver unless administered in a long-acting injectable (LAI) form. It is metabolized by dehydrogenation, hydroxylation, and *N*-dealkylation. *In vitro* studies show that CYP3A4 and CYP2D6 conduct the dehydrogenation and hydroxylation of aripiprazole, with CYP3A4 additionally catalyzing the *N-*dealkylation. Although a substrate for these enzymes, it does not appear to inhibit the activity of these enzymes. In clinical studies, 10–30 mg/day doses of aripiprazole had no significant effect on the metabolism of substrates of CYP2D6 or CYP3A4 activity as indexed by dextromethorphan; it does not appear to be an inhibitor of CYP2C9, CYP2C19, or CYP1A2 (Hjorthoj et al., 2015), nor a substrate for CYP1A enzymes, and hence no dose adjustment is required in smokers.

In a large pharmacokinetic study (*N* = 1288), CYP2D6 PMs and IMs had a 1.4 times increase in exposure to the active moiety compared to NMs, leading to a 15% decrease in medication dosage of aripiprazole. Switch in medication from aripiprazole was not, however, significantly associated with CYP2D6 status (Jukic et al., 2019).

The active dehydro-aripiprazole metabolite has a similar affinity as aripiprazole for dopamine D_2_ receptors; at steady state it represents about 40% of the plasma concentration of aripiprazole (area under the curve or AUC; Hjorthoj et al., 2015), after oral administration or 29–33% after administration in the form of the LAI Abilify Maintena, and is therefore thought to contribute to the sustained pharmacologic effect of aripiprazole. Both aripiprazole and dehydro-aripiprazole are highly bound to plasma protein, mainly to albumin (reviewed in DeLeon et al., 2004). The average elimination half-life is oral aripiprazole is ∼75 h, but in CYP2D6 PMs, the average half-life extends to ∼146 h (Hjorthoj et al., 2015). The half-life of oral aripiprazole in CYP2D6 IMs (75.2 h) was significantly longer than that in CYP2D6 NMs (45.8 h); the systemic clearance of aripiprazole in IMs is approximately 60% that of NM subjects, with the maximum concentration being the same in IMs as in NMs (Kubo et al., 2007). At steady state, PMs have a significantly lower concentration to dose ratio than NMs, while in one report, IMs did not differ (van der Weide and van der Weide, 2015). However, van der Weide and van der Weide (2015) included in their IM group individuals who were heterozygous NMs (NM/PM genotype). In another report (Hendset et al., 2014), median serum concentrations were 1.6-fold or 1.8-fold higher in individuals of CYP2D6 PM/IM or IM/IM genotype, respectively than in those who were heterozygous NMs.

For patients who are known CYP2D6 PMs, FDA recommends administration of half of the usual dose of aripiprazole, and the DPWG guidelines recommend reducing maximum daily dose to 10 mg/day or 300 mg/month, i.e., 67–75% of the standard maximum dose.^15^ Given that at a dose as low as 2 mg, D_2_ receptor occupancy is ∼70% (71.6 ± 5.5%, Kegeles et al., 2008), and the recommendation by consensus guidelines of doses of aripiprazole lower than those used in the initial marketing phase of the drug (Aitchison et al., 2009), it may well be recommendable to start at the lowest dose (2 mg) and to go no higher than 5 mg in CYP2D6 poor metabolizers. While there are as yet no guidelines for other CYP2D6 phenotypic groups, in the case of IMs, the Japanese data would suggest that a cautious dosing in the 2–5 mg range should be appropriate.

In addition, packaging information for aripiprazole offers additional guidelines should the medication be taken with known CYP inducers or inhibitors (Abilify - aripiprazole tablet, 2016). In the case of co-prescription of CYP3A4 or CYP2D6 inhibitors, dosage is recommended to be reduced (by 50% in the case of strong inhibitors such as ketoconazole and fluoxetine, respectively). Likewise, should aripiprazole be taken with known CYP3A4 inducers, dosage increase is recommended (doubling in the case of carbamazepine). On cessation of any inhibitors/inducers, the dose should be readjusted accordingly (Abilify).^16^

It has been noted that aripiprazole and 2,3-(dichlorophenyl) piperazine (2,3-DCPP), one of its metabolites, affect cholesterol biosynthesis by inhibiting 7-dehydrocholesterol reductase (DHCR7), the enzyme that converts 7-dehydrocholesterol (7-DHC) to cholesterol (Korade et al., 2010, 2016; Kim et al., 2016; Genaro-Mattos et al., 2018). Cholesterol is of critical importance to brain development; mutations in *DHCR7* gene leads to Smith-Lemli-Opitz Syndrome, a neurodevelopmental condition, and exposure to DHCR7 inhibitors during the first trimester of pregnancy is associated with increased rates of fetal malformations, intrauterine death, and spontaneous abortions (Boland and Tatonetti, 2016). Thus, aripiprazole should be contraindicated during the first trimester of pregnancy; the Summary of Product Characteristics states^17^ “this medicinal product should not be used in pregnancy unless the expected benefit clearly justifies the potential risk to the fetus.” The most critical period for formation of the neural tube is the first six weeks of gestation, when many women do not realize they are pregnant. Therefore, it is recommended that women receiving aripiprazole in reproductive years should have a discussion of whether the woman is sexually active and of methods of contraception.

In a multiple-dose study, the mean terminal-phase elimination half-life of aripiprazole was 29.9 days and 46.5 days after 4-week injection of LAI 300 mg dose and 400 mg dose, respectively (Mallikaarjun et al., 2013). Data regarding differential half-life of the LAI by CYP2D6 genotype and/or CYP3A activity are not available. Aripiprazole lauroxil is a prodrug that undergoes bioactivation by hydroxylation and can be administered once every 6 weeks; it is similarly lacking pharmacogenetic data thus far.

#### Risperidone

Risperidone is an atypical antipsychotic used for treating schizophrenia, acting mainly on 5-HT_2A_ and D_2_ receptors (Zhou et al., 2009); it is converted to 9-hydroxyrisperidone (9-OH-RIS) by CYP2D6, with the latter being excreted in the urine and feces. In a meta-analysis conducted by Stingl and Viviani (2015), they estimated dose adjustment of RIS to be 56 and 146% in CYP2D6 PMs and UMs, respectively, mentioning increased risk of toxicity in PMs. The DPWG note increased risk of treatment failure in CYP2D6 PMs and UMs and recommend using 67% of the standard dose in the former, and choosing an alternative drug or titrating the dose according to the maximum for the active metabolite (12 mg/day of paliperidone) in the latter. In a recent review by van Westrhenen et al. (2020), these recommendations were updated to suggest reducing the maximum dose by 33% (to 4 mg/day) in IMs as well as in PMs. For UMs, it was suggested to select an alternative medication or use therapeutic drug monitoring. It is worth noting that Cui et al. (2020) have found significant differences in recommendations of RIS dosage according to ethnicity. Specifically, adjustment in titration of this medication should be reduced in people of Asian ethnicity compared to Whites.

In a Norwegian population (Mannheimer et al., 2014, 2016), it was found that the metabolic ratio (MR) for RIS, expressed as RIS/9-OH-RIS, was, not surprisingly, associated with CYP2D6 PM status: an MR threshold of >1 predicted PM status with 91% accuracy (Mannheimer et al., 2016).

RIS metabolism by CYP2D6 is inhibited by the phenothiazine drug perazine when the two are co-administered (Paulzen et al., 2017), resulting in an increase in RIS and (RIS + 9-OH-RIS) concentrations and a reduction in the 9-OH-RIS/RIS ratio. Animal models have previously shown the role of phenothiazines in inhibiting the CYP2D family (Daniel et al., 2005).

In a study focusing on the relationship between genetic and epigenetic variation and response to RIS, three CpG sites in CYP2D6 and two to three CpG sites in CYP3A4 appeared to be more methylated in poor responders (Shi et al., 2017).

The effect of *CYP2D6* genotype on RIS metabolism has been studied in young Thai autistic spectrum individuals (Nuntamool et al., 2017). Genotypes *CYP2D6^∗^5/^∗^10*, *^∗^10/^∗^10* and *^∗^10/^∗^41* showed reduced RIS metabolism, with significantly higher RIS plasma concentrations. While such an association was not seen in the *CYP2D6^∗^4/^∗^10* genotype group, this was likely owing to the relatively low frequency of the *CYP2D6^∗^4* variant in this ethnicity, and the wide spread of the data in the small subgroup of *CYP2D6^∗^4/^∗^10* genotype. The *CYP2D6^∗^10* variant was also associated with higher MR of RIS/9-OH-RIS. Hendset et al. (2014) genotyped for *CYP2D6 ^∗^3*, *^∗^4,^∗^ 5*, *^∗^6*, *^∗^9*, *^∗^10* and *^∗^41*, and classified as “*^∗^1/def*” (heterozygous for normal and deficient function) or “*def/red*” (heterozygous for deficient and reduced function); RIS serum concentration was 4.5 times higher in the *def/red* group compared to *^∗^1/def*. In addition, a 3 to 4-fold increase in the serum concentration of RIS was shown in the *red/red* group.

In addition to variation in metabolic activity and treatment response, Molden et al. (2016) found evidence of a relationship between genotype and discontinuation of treatment. Individuals classified as PMs for *CYP2D6* had active moiety (RIS + 9-OH-RIS) concentration 1.5 times higher than NMs. Consequently, there was an over-representation of adverse events and discontinuation of treatment for PMs. Conversely, a similar study with Croatian psychiatric patients receiving RIS injections found individuals classified as UM with concentrations of RIS active moiety (RIS + 9-OH-RIS) not reaching the threshold recommended for therapeutic range (Ganoci et al., 2016). Kaur et al. (2017) reported an association between the *CYP2D6^∗^4* PM haplotype and treatment dropout due to poor response.

In the largest study of RIS and CYP2D6 to date (1288 patients), approximately 1.4 and 1.6-fold RIS exposure increase was observed in CYP2D6 IMs and PMs, respectively (Jukic et al., 2019). A higher incidence of RIS-associated ADRs (de Leon et al., 2005) and treatment failure (Jukic et al., 2019) is observed in CYP2D6 PMs compared with NMs, with increased treatment failure rate also being observed in CYP2D6 UMs (Jukic et al., 2019). It is possible that the latter may be exposed to subtherapeutic drug concentrations, and also possible the effect of CYP2D6 on normally minor synthesis pathways for serotonin and dopamine may at least partly relate to such associations. Recent systematic reviews and meta-analyses support the need for dosage adjustment of RIS based on *CYP2D6* genotype (Cui et al., 2020; Zhang et al., 2020).

The relationship between CYP2D6 and hyperprolactinemia (a possible adverse effect of RIS) appears to be U-shaped, with a tendency (though not consistently replicated) for both extremes of CYP2D6 metabolic phenotype (i.e., PMs and UMs) to show an association with hyperprolactinemia (Troost et al., 2007; Roke et al., 2013; Youngster et al., 2014; Sukasem et al., 2016). Hyperprolactinemia has also been associated with the *DRD2* Taq1A variant (Sukasem et al., 2018).

Interaction of known CYP2D6 inhibitors such as fluoxetine, bupropion, lamotrigine, sertraline, and citalopram are strongly correlated with the concentration of RIS in young male patients, compared to the available concentration of its metabolites (Calarge and Miller, 2011). A similar relationship has been described for thioridazine, and levomepromazine (Mannheimer et al., 2008). The same relationship was not found for duloxetine, another known CYP2D6 inhibitor (Hendset et al., 2010). Ishak et al. (2008) have described an association between RIS discontinuation caused by DDI from CYP2D6 inhibitors.

To a lesser extent, RIS metabolism is also mediated by CYP3A4 and DDI with inducers of this CYP enzyme are supported by the literature. Co-medication with armodafinil results in a decrease in plasma concentration of both RIS and 9OH-RIS (Darwish et al., 2015). The same relationship is true for rifampin (Mahatthanatrakul et al., 2007; Kim et al., 2008).

#### Olanzapine

Olanzapine is an antipsychotic licensed for use in schizophrenia and related psychotic disorders and bipolar disorder. The main circulating metabolites are desmethylolanzapine and olanzapine-10-glucuronide (Callaghan et al., 1999; Erickson-Ridout et al., 2011; Lu et al., 2016). The conversion to desmethylolanzapine is predominantly catalyzed by CYP1A2, with lesser roles for CYP2D6, CYP2C8, and CYP2C19 (Ereshefsky, 1996; Callaghan et al., 1999; Korprasertthaworn et al., 2015; Okubo et al., 2016). Okubo and colleagues investigated the role of CYP1A2, CYP2D6, and FMO3 in individuals of varying *CYP2D6* and *FMO3* genotype (Okubo et al., 2016). Olanzapine *N*-demethylation and *N*-oxygenation were found to be catalyzed by CYP1A2 and CYP2D6, and by CYP2D6 and FMO3, respectively, in experiments using liver microsomes and recombinant enzymes. The effects on olanzapine oxidation activities of furafylline (which inhibits CYP1A2), quinidine (inhibits CYP2D6), and heat treatment (inhibits FMO3-mediated activities) were investigated. Each, and the combination of all three treatments suppressed the metabolic clearances of olanzapine by 28, 33, 25, and 85%, respectively. Using recombinant CYP2D6 enzymes CYP2D6.1 and CYP2D6.10, only the wild-type variant was capable of the 2-hydroxylation conversion of olanzapine into 2-hydroxymethyl-olanzapine; CYP2D6 appears to be the only enzyme catalyzing olanzapine 2-hydroxylation. Direct glucuronidation (at the 10 and 4 positions) is conducted by UGT1A4 and UGT2B10, with the *UGT1A4^∗^3* and *UGT2B10^∗^2* haplotypes being associated with increased and decreased glucuronidation, respectively (Erickson-Ridout et al., 2011).

Drug-drug (Drug Interactions Flockhart Table^TM^; DrugBank) interactions are of importance in the prescription of olanzapine. CYP1A2 is induced by smoking; the plasma concentration to dose ratio of olanzapine is therefore lower in smokers (Tsuda et al., 2014). Inhibition of CYP1A2 by fluvoxamine also increases the concentration to dose ratio (Chiu et al., 2004). CYP1A2 is also inhibited by estrogens; as a result, gender (clearance is reduced in women) and body fat content influence the metabolism of olanzapine (Ereshefsky, 1996; Callaghan et al., 1999). Valproic acid co-prescription results in a decrease in olanzapine concentration (reviewed by Vella and Mifsud, 2014). In contrast, protease inhibitors used in the treatment of HIV such as ritonavir in combination with fosamprenavir induce olanzapine metabolism (via CYP1A2 and/or UGT), leading to a recommendation to increase olanzapine dose by 50% when prescribed with such (Jacobs et al., 2014).

#### Quetiapine

Quetiapine is another commonly prescribed antipsychotic. While literature supports CYP3A4 being the main enzyme in the quetiapine metabolic pathway, CYP2D6 is involved in the further metabolism of its principal metabolite, *N*-desalkylquetiapine. In an analysis of therapeutic drug monitoring data, patients from a Norwegian psychiatric hospital were genotyped for *CYP2D6*, *CYP3A5*, and *ABCB1* (3435C>T) and the associations with dose-corrected serum concentrations of quetiapine and *N-*desalkylquetiapine were analyzed (Bakken et al., 2015). The mean dose-corrected serum concentration (C/D) of *N*-desalkylquetiapine was estimated to be 33 and 22% higher in CYP2D6 PMs (*P* = 0.03) and heterozygous CYP2D6 NMs (*P* = 0.001), respectively, compared with CYP2D6 NMs. There was no significant association with *ABCB1* 3435C>T polymorphism or *CYP3A5* genotype.

Quetiapine has, however, been observed to have a serum level 2.5 times higher in those either heterozygous or homozygous for *CYP3A4^∗^22* compared to those of *CYP3A4* wild-type (van der Weide and van der Weide, 2014), with concentration to dose ratios that were 67% higher. The percentage of patients who had levels of quetiapine above the therapeutic range was also about five times higher in the *^∗^22* carrier group (16.1 versus 2.9%). Quetiapine serum levels based on reduced CYP3A4 metabolic activity were comparable to results found with coadministered CYP3A4 inhibitors, such as ketoconazole. The frequency of the *CYP3A4^∗^22* haplotype is up to 10%.

In terms of DDI, valproate coadministration with quetiapine appears to result in a variable degree of increase in quetiapine plasma levels (Aichhorn et al., 2006; Winter et al., 2007), which may result in toxicity on occasion (Anderson and Fritz, 2000). In a review of therapeutic monitoring data from more than 2000 patients, it was reported that the following factors were associated with an increase in quetiapine concentration: age of at least 70 years, comedication with clozapine, fluvoxamine, and to a lesser extent citalopram/escitalopram, while, conversely, the following were associated with reduced quetiapine concentration: age under 18 years and comedication with carbamazepine or oxazepam, and to a lesser extent levomepromazine or lamotrigine (Castberg et al., 2007). The largest effect sizes were seen with fluvoxamine (+159%), clozapine (+82%), age at least 70 years (+67%), and carbamazepine (−86%). Another study found that dose-corrected quetiapine concentrations were approximately 60% lower in patients co-medicated with lamotrigine (Andersson et al., 2011).

#### Ziprasidone

Ziprasidone is a less commonly prescribed antipsychotic. Data indicate ziprasidone is mainly metabolized by glutathione and enzymatic reduction by aldehyde oxidase, followed by *S*-methylation to *S*-methyl-dihydroziprasidone by thiolmethyltransferase (Obach and Walsky, 2005). Approximately one third of its clearance is thought to be CYP3A4-dependent (Beedham et al., 2003). It is therefore subject to CYP3A-mediated induction (e.g., by carbamazepine, Miceli et al., 2000) and inhibition effects. As this medication has been associated with increases in the QT_*c*_ interval (Aronow and Shamliyan, 2018), inhibition effects (e.g., by fluvoxamine or ketoconazole) should be avoided. Ziprasidone is also contraindicated in the presence of other medications that also prolong QT_*c*_ (Kutcher et al., 2005; Table 4 in Hicks et al., 2017).

### Antidepressants

Tricyclic antidepressants (TCAs) and SSRIs both undergo first pass metabolism in the liver, with the CYP enzymes playing a prominent role in this. The cytochromes involved include CYP2D6, CYP2C19, CYP2C18, the CYP3A family, CYP1A2, CYP2C9, and CYP2B6, with the first two enzymes having a higher affinity for most antidepressants than the rest of the enzymes (Brosen, 1993; Koyama et al., 1997; Jann and Cohen, 2000).

#### Tricyclic Antidepressants

Imipramine, the first TCA was derived from a phenothiazine, showing improvement without serious side effects in 500 patients with severe depression (Kuhn, 1958; Hillhouse and Porter, 2015). Although TCAs are still used (e.g., second-line or with somatic symptoms) to treat depression (Uher et al., 2009b; American Psychiatric Association, 2010; Kennedy et al., 2016), the treatment of pain (e.g., migraine, neuropathic, cancer-associated) is now their more common therapeutic use (Watson, 2000; Laird et al., 2008; Baltenberger et al., 2015).

Tricyclics include tertiary and secondary amines. A tertiary amine has a nitrogen bonded to three carbons, while in the case of a secondary amine, the nitrogen is bonded to only two carbons. The tricyclics amitriptyline, clomipramine, imipramine, trimipramine, doxepin, and dothiepin are tertiary amines. Tertiary amines are demethylated to secondary amines mainly by CYP2C19, but also by CYP1A2, CYP2C9, and CYP3A4, while both tertiary and secondary undergo parallel hydroxylation reactions mainly by CYP2D6, with CYP2C19 making a lesser contribution (Bertilsson et al., 2002; Aitchison, 2003; Bertilsson, 2007). The secondary amine metabolites of amitriptyline and imipramine are nortriptyline and desipramine, respectively, each also available as licensed medications.

Using hepatic microsomes of varying CYP2C19 activity and recombinant CYPs, Koyama et al. (1997) demonstrated that imipramine *N*-demethylation was catalyzed by CYP2C19 and CYP1A2 (high affinity and low affinity components, respectively), imipramine 2-hydroxylation was mediated by CYP2D6 and CYP2C19 (high affinity and low affinity components, respectively), and that in individuals deficient in CYP2C19, CYP1A2, and CYP2D6 play a major role in imipramine *N*-demethylation and 2-hydroxylation respectively. Among the recombinant human CYPs, CYP2C19, 2C18, 2D6, 1A2, 3A4, and 2B6 in rank order catalyzed the *N*-demethylation, whereas CYP2D6, 2C19, 1A2, 2C18, and 3A4 catalyzed the *2*-hydroxylation. In a monoclonal antibody inhibition, Yang et al. (1999) concluded similarly that imipramine was metabolized to 2-hydroxyimipramine by 2C19 and 2D6, and to desipramine by 1A2, 2C18, 2C19, and 2D6, with the contributions of the isoforms to desipramine formation varying for 2C19 (13–50%), 1A2 (23–41%), and 3A4 (8–26%).

Tricyclic antidepressants inhibit presynaptic noradrenaline (also known as norepinephrine) and serotonin reuptake via the noradrenaline and serotonin transporters, respectively, with the tertiary amines having a greater affinity for the serotonin transporter than the secondary amines, which are relatively selective for the noradrenaline transporter (Owens, 1996). The tertiary amines are therefore dual SNRIs, while the secondary amines are noradrenaline reuptake inhibitors (or NARIs). There are also contrasts in their CYP inhibition. Tertiary amines TCAs (e.g., amitriptyline, imipramine) inhibit CYP2C19 (estimated Ki of 37.7 and 56.8 μM, respectively). By contrast, the secondary amines show negligible CYP2C19 inhibition activity (Shin et al., 2002) but inhibit CYP2D6 slightly more than tertiary amine TCAs; for example, estimated K_*i*_ values for the tertiary amine TCAs amitriptyline and imipramine are 31.0, 28.6 μM, respectively, with K_*i*_ s for nortriptyline and desipramine being 7.9 and 12.5 (Shin et al., 2002). Although therapeutic plasma concentrations are less than 1 μM (Yau et al., 2007; Aitchison et al., 2010), cerebral concentrations may be higher (Weigmann et al., 2000; Aitchison et al., 2010). Further, this differential may be affected by factors such as p-gp expression at the blood–brain barrier. It is therefore possible that with repeated dosing, as the concentration of a tertiary amine TCA increases in the brain, that the level of CYP2C19 inhibition increases, and that this leads to reduction in the demethylation reaction centrally. This would be expected to be associated with a greater degree of dual reuptake inhibition and may at least partly explain the clinical observation of time for antidepressant effect to maximize. This hypothesis is consistent with a report of an inverse relationship between CYP2C19 activity and response to TCAs (mainly tertiary amines, Aitchison et al., under revision).

There are also contrasts between the tertiary and secondary amines and side effect/adverse drug reaction potential. The side effects are associated with antagonism at the following receptors: adrenergic α1 and α2 receptors, muscarinic (cholinergic) receptors, and histamine H1 receptors (Cusack et al., 1994; Owens et al., 1997; Sanchez and Hyttel, 1999). Specifically, blockade of muscarinic receptors in the parasympathetic nervous system is associated with dry mouth, blurred vision, constipation, urinary retention, and if at toxic levels, delirium; alpha adrenergic receptor antagonism is associated with orthostatic hypotension; and histamine H1 receptor blockade with sedation and weight gain. Other side effects (such as palpitations, vertigo, sweating, tremors, and interference with sexual function) may also occur (Asberg et al., 1970; Ziegler et al., 1978; Uher et al., 2009a; Hodgson et al., 2015) and may represent more than one pharmacodynamic mechanism. The tertiary amine TCAs have greater cholinergic receptor binding than the secondary amines, which in turn have greater affinity than the hydroxylated metabolites (Rudorfer and Potter, 1999). Some effects may be related to specific metabolites (e.g., *N*-methyl quaternary ammonium derivatives of amitriptyline, doxepin, and imipramine are antagonists at both central nervous system and cardiac muscarinic receptors) (Ehlert et al., 1990). Hydroxylated metabolite concentration has been associated with increases QTc interval (Schneider et al., 1988; Stern et al., 1991). It is therefore possible that CYP2D6 UM might have elevated hydroxy-metabolite plasma concentrations (Bertilsson et al., 1985) resulting in an increased risk of cardiotoxicity. Moreover, therapeutic drug monitoring does not usually include measuring hydroxylated metabolite plasma concentrations. In the case of a combination of CYP2C19 poor metabolizer status and CYP2D6 UM status, it might be advisable to avoid TCA prescription; this is in fact the CPIC recommendation for this combination [Table 4 in Hicks et al. (2017)].

The association between *CYP2D6* and *CYP2C19* genotype and clinical response to TCAs (treatment efficacy and/or side effects) has been reviewed (Hicks et al., 2017). In brief, studies support the existence of a concentration–effect relationship for TCAs and/or their active metabolites (Ziegler et al., 1977; Sjöqvist et al., 1980; Gram et al., 1984; Perry et al., 1987) (Preskorn and Jerkovich, 1990). In an early report, high concentrations of nortriptyline were linked to adverse effects, with decreased antidepressant effect (Asberg et al., 1970). Concentration-dependent side effects have been observed in individuals deficient in CYP2D6 when treated with usual doses of TCAs from accumulation of the parent drug and/or active metabolites (Bertilsson et al., 1981; Sjöqvist and Bertilsson, 1984; Balant-Gorgia et al., 1989). Ethnic groups with a higher frequency of CYP2D6 IM alleles achieve higher levels of TCAs than Whites and have a faster rate of recovery from depressive episodes (Raskin and Crook, 1975; Ziegler and Biggs, 1977; Rudorfer and Robins, 1982). An excess of CYP2D6 PM alleles has been found in amongst patients with a history of adverse reactions to TCAs and relevant SSRIs (Chen et al., 1996). CYP2D6 PMs have high levels of desipramine, associated with adverse effects necessitating dose reduction (Spina et al., 1997). An inverse correlation between the frequency of adverse drug events and number of functional CYP2D6 genes has been found, including patients on TCAs (Chou et al., 2003). Studies published since the Hicks et al. (2017) review include that of Hodgson et al. (2015).

There are guidelines by both CPIC and DPWG (for a comparison, see Bank et al., 2018). CPIC guidelines for CYP2D6 are as follows: for both PMs and UMs, it is suggested to avoid use of TCAs due to possible side effects or subthreshold concentrations, respectively. In both cases, when TCAs are still prescribed, therapeutic drug monitoring is recommended, with PMs starting at 50% regular dosage and for UMs consideration being given to use TDM to titrate up to a higher target dose. The DPWG provide specific suggested increases in the starting dosages for amitriptyline, clomipramine, doxepin, imipramine, and nortriptyline of 125, 150, 200, 170%, respectively followed by TDM (Bank et al., 2018). For CYP2D6 IMs, a 25% reduction in the initial dose is recommended by CPIC. For many drugs, evidence is still accumulating, and therefore implementation of the recommendations is “optional,” or at prescriber discretion.

In regard to CYP2C19 status, there are CPIC guidelines for the tertiary amines amitriptyline, clomipramine, doxepin, imipramine, and trimipramine. For CYP2D6 UMs, RMs, or PMs, CPIC provides an optional recommendation to substitute with medications not metabolized by CYP2C19, such as nortriptyline or desipramine. In the case of CYPC19 PMs, a 50% decrease in initiation dose is suggested, with TDM to guide titration (Hicks et al., 2017).

There are also CPIC guidelines for amitriptyline where both CYP2D6 and CYP2C19 data are available. If an individual is a CYP2D6 or CYP2C19 PM and a NM for the other enzyme, it is recommended to avoid the medication or, if warranted, consider a 50% decrease in initiation dose; for a CYP2D6 UM and CYP2C19 NM, it is recommended to avoid the medication or, if warranted, consider titrating to a higher target dose (compared to CYP2D6 NMs); and for a CYP2D6 IM and CYP2C19 NM, to consider a 25% decrease in initiation dose ((Hicks et al., 2017). No adjustments in dosage is necessary for those who are NMs for CYP2D6 and CYP2C19, or an NM for CYP2D6 and an IM for CYP2C19 (Hicks et al., 2017).

#### Tetracyclic Antidepressants

Mirtazapine acts as antagonist at adrenergic α_2_-autoreceptors and α_2_-heteroreceptors as well as at 5-HT_2_ and 5-HT_3_ receptors (Anttila and Leinonen, 2001). The α_2_-autoreceptor blockade leads to increased release of noradrenaline while the blockade of α_2_-heteroreceptor on serotonergic neurons increases serotonin release. Owing to antagonism of 5-HT_2_ and 5-HT_3_, transmission is enhanced at only 5-HT_1A_ (and related receptors). It is a racemic mixture of *R*(−) and *S*(+)-enantiomers, with effects on heart rate and blood pressure correlating more strongly with *R* (−) than with *S* (+) concentration, and sedation being associated with both enantiomers (Brockmöller et al., 2007). The main metabolic pathway for mirtazapine is 8-hydroxylation, catalyzed by mainly by CYP2D6 (65%) at low concentrations, reducing to 20% at higher concentrations, where CYP1A2 (50%), CYP3A4 (20%), and CYP2C9 (10%) contribute more (Dahl et al., 1997; Störmer et al., 2000). Other metabolic pathways are *N*-demethylation and *N*-oxidation. The former is conducted by mainly by CYP3A4 (50–70%), with CYP1A2 (50% at low concentrations, 5% at high concentrations), CYP2C8 (<20%), and CYP2C9 (<5%) also contributing. *N*-oxidation is catalyzed by CYP1A2 and CYP3A4, with the former playing a larger role (80%) at low concentrations and the latter being responsible for a greater proportion (85%) of the reaction at higher drug concentrations (Dahl et al., 1997; Störmer et al., 2000). Enzyme polymorphism may additionally affect the relative contributions of these CYPs.

The maximum concentration and area under the curve are greater in females as compared to males (Timmer et al., 2000; Sennef et al., 2003). In non-smokers and at lower concentrations of mirtazapine, *CYP2D6* genotype affects the plasma levels and clearance of the *S*-enantiomer and its metabolites (Störmer et al., 2000; Brockmöller et al., 2007; Sirot et al., 2012; Hayashi et al., 2015). At higher concentrations (250 μM), CYP3A4 contributes to about 70%, while CYP2D6, CYP2C8, CYP2C9, and CYP1A2 each account for less than 15% of its metabolism (Störmer et al., 2000). Unlike the tricyclics, there is no clear relationship between mirtazapine plasma concentration and its efficacy. While an increase in the maximal serum concentration for coadministered amitriptyline has been described (Sennef et al., 2003), the overall inhibitory effect of mirtazapine on CYPs is not thought to be clinically significant (Anttila and Leinonen, 2001; Spina et al., 2008). In the Sennef et al. (2003) study, coadministered amitriptyline increased the maximum concentration of mirtazapine (by 36%) in only males. *S*-hydroxymirtazapine concentration has been reported as being elevated in individuals of *CYP2B6^∗^6/^∗^6* genotype (Sirot et al., 2012). As yet, there are no DPWG or CPIC guidelines for mirtazapine based on genotype.

#### Selective Serotonin Reuptake Inhibitors

The second SSRI to be synthesized, fluoxetine was the first SSRI to enter widespread use (Wong et al., 1974, 1975). Selective serotonin reuptake inhibitors are now widely used to treat depression, escitalopram having the highest affinity for the serotonin transporter (Owens et al., 2001) and being an allosteric modulator, one molecule increasing the binding of a second at this target.

In brief, SSRIs are partly metabolized by CYP2D6 (Brosen, 1993). Demethylenation is the initial step of paroxetine metabolism (an SSRI), primarily conducted by CYP2D6 (a high affinity saturable process,Bloomer et al., 1992). Further conjugation of paroxetine results in glucuronide and sulfate conjugated metabolites (Haddock et al., 1989). Paroxetine is a potent competitive inhibitor of CYP2D6 (Bourin et al., 2001) nonetheless, differences in steady-state plasma concentration of paroxetine by CYP2D6 phenotype are seen (Gex-Fabry et al., 2008). Higher doses of paroxetine (e.g., 30 mg) are associated with a greater degree of CYP2D6 inhibition (Findling et al., 2006). In diabetic neuropathy, paroxetine has an analgesic effect, plasma concentrations greater than 300–400 nmol/l being associated with optimal response (Sindrup et al., 1990, 1991).

Fluoxetine is a racemic mixture of *S*(+) and *R*(−)-fluoxetine, with the former being metabolized predominantly by CYP2D6 to *S*-norfluoxetine and the latter by CYP2D6 and CYP2C9 to *R*-norfluoxetine; CYP3A4 and CYP2C19 make minor contributions to this demethylation reaction (Hicks et al., 2015). *R/S*-fluoxetine and *S*-norfluoxetine are all potent SSRIs, with *R*-norfluoxetine being 20 times less potent (Hicks et al., 2015). The strength of CYP2D6 inhibition for SSRIs is as follows in reducing order: paroxetine, fluoxetine, norfluoxetine, desmethylcitalopram, fluvoxamine, sertraline, citalopram (Baumann and Rochat, 1995). Although fluoxetine is less potent as an inhibitor of CYP2D6 than paroxetine, owing to its substantially longer half-life – 1–3 days and 4–6 days after acute and chronic administration, respectively, with the corresponding values being 4 and 16 days for norfluoxetine,^18^ inhibition effects may endure for weeks to months after multiple dosing (Liston et al., 2002). Fluoxetine and sertraline also inhibit CYP2C19 (Bertilsson and Dahl, 1996) while norfluoxetine is a moderate CYP3A4 inhibitor (Hemeryck and Belpaire, 2002).

The primary route of metabolism for citalopram (a racemic mixture of the *R*- and *S*-enantiomers of citalopram) and escitalopram (*S*-citalopram) is *N*-demethylation by CYP2C19, CYP2D6, and CYP3A4 (Sindrup et al., 1993; Kobayashi et al., 1997; Rochat et al., 1997; von Moltke et al., 2001). CYP2D6 then conducts the *N*-demethylation of *N*-desmethylescitalopram to *N*-didesmethylescitalopram (von Moltke et al., 2001). The medication and its metabolites may inhibit enzymes: citalopram and *R*- or *S*-desmethylcitalopram are weak inhibitors of CYP2C19, while *R*- and *S*-didesmethylcitalopram are moderate inhibitors, with mean IC_50_ values of 18.7 and 12.1 μM, respectively. *S*-citalopram and *S*-desmethylcitalopram are weak inhibitors of CYP2D6 (IC_50_ = 70–80 μM); *R*-desmethylcitalopram shows stronger inhibition at this enzyme (IC_50_ 25.5 ± 2.1 μM) (von Moltke et al., 2001). Fluvoxamine is predominantly a CYP1A2 inhibitor (Christensen et al., 2002) but also inhibits other CYPs including the CYP3As (Hemeryck and Belpaire, 2002).

SSRIs are more 20 to 1500-fold more selective for inhibiting serotonin than noradrenaline. They do not stimulate the release of serotonin or norepinephrine presynaptically (Rothman et al., 2001) and have weak/no-direct pharmacological action at postsynaptic serotonin receptors (e.g., 5-HT_1A_, 5-HT_2A_, and 5-HT_2C_) (Thomas et al., 1987; Owens et al., 1997; Sanchez and Hyttel, 1999), and minimal binding affinity for other postsynaptic receptors (adrenergic α_1_, α_2_, and β, histamine H_1_, muscarinic, and dopamine D_2_ receptors) (Thomas et al., 1987; Owens et al., 1997).

Associations between SSRI phenotypes (concentrations, efficacy, tolerability) and *CYP2D6* and *CYP2C19* genotypes are provided in Supplementary Tables S7–S11 in Hicks et al. (2015). In a meta-analysis of the main functional *CYP2C19* variants in Whites (the *CYP2C19^∗^2* and the *CYP2C19^∗^17*, plus wild-type by exclusion of these) for individuals treated with citalopram or escitalopram (in the GENDEP, STAR^∗^D, GenPod, and PGRN-AMPS studies), CYP2C19 PMs had greater symptom improvement and higher remission rates compared to NMs (Fabbri et al., 2018). This is consistent with earlier data indicating that CYP2C19 PMs respond better to escitalopram if treatment is tolerated (Mrazek et al., 2011). At weeks 2–4, PMs showed increased risk of side effects (Fabbri et al., 2018). In a retrospective analysis of data from 2087 patients treated with escitalopram and genotyped for *CYP2C19*, PMs had an increase in exposure and a higher rate of treatment dropout compared with CYP2C19 NMs (Jukic et al., 2018). Conversely, the *CYP2C19^∗^17* haplotype was associated with an increase in CYP2C19 activity by approximately 20%, with those of *CYP2C19^∗^1/^∗^17* and *CYP2C19^∗^17/^∗^17* genotype showing a 50% increase in treatment failure rate compared with NMs (Jukic et al., 2018). Moreover, replicated findings that CYP2C19 UMs treated with escitalopram exhibit increased suicidal ideation (Jukic et al., 2017; Rahikainen et al., 2019) indicates that distinguishing between CYP2C19 NMs and UMs is clinically relevant for the escitalopram treatment. Shelton et al. (2020) using a combinatorial PGx algorithm (covering several different genes) reported a significant association with variation in the metabolism of escitalopram/citalopram.

The CPIC guidelines for SSRIs (Hicks et al., 2015) cover two medications for *CYP2D6*: paroxetine and fluvoxamine. The recommendation for paroxetine in the case of CYP2D6 UMs is to select an alternative drug and likewise for PMs, with implementation being optional for the latter. For fluvoxamine, in the case of CYP2D6 UMs there was insufficient data for a recommendation, with an optional recommendation to consider a 25–50% reduction in the starting dose for CYP2D6 PMs, and titrate to response, or consider using an alternative medication not metabolized by CYP2D6.

Three medications are included in the CPIC SSRI guidelines in regard to *CYP2C19*: citalopram, escitalopram and sertraline. A 50% reduction of the standard dosage for the three drugs is recommended for PM status, with and titration to response, or considering using an alternative medication not metabolized by CYP2D6 (strength of the recommendation being moderate for citalopram and escitalopram and moderate for sertraline). For CYP2C19 UMs, for citalopram and escitalopram, selection of a medication not metabolized by CYP2C19 is recommended, while for sertraline, initiation at the normal dose may be tried, with substitution being considered if patients do not respond to treatment. The recommendations are classified as moderate for citalopram/escitalopram and optional for sertraline (Hicks et al., 2015).

In addition to the CPIC guidelines, Stingl et al. (2013) suggest that in the case of fluoxetine (not included in the guidelines above), due to its role as both substrate and inhibitor of CYP2D6, physicians should be careful if co-prescribing it with other CYP2D6 substrates.

#### Serotonin Noradrenaline Reuptake Inhibitors

Venlafaxine is a SNRI, which means that like the tertiary amine tricyclics, it inhibits neurotransmitter reuptake at both the serotonin and the noradrenaline (also known as epinephrine) transporters The major metabolic route for venlafaxine is *O*-demethylation, which is mediated very specifically by CYP2D6 to an active metabolite, *O*-desmethylvenlafaxine (Otton et al., 1996). The *N*-demethylation is conducted by CYP3A4 and CYP2C19 (Sangkuhl et al., 2014). This means that the ratio of the *O*- and *N*-demethylated metabolites of venlafaxine may in fact be used as a biomarker of CYP2D6 activity, predicting CYP2D6 poor metabolizers with a specificity and sensitivity of >85% (Mannheimer et al., 2016). In *in vitro* studies, venlafaxine is a weaker inhibitor of CYP2D6 than are the SSRIs paroxetine, fluoxetine, fluvoxamine, and sertraline, and has minimal or no effect on CYP1A2, CYP2C9, and CYP3A4 (Ball et al., 1997; von Moltke et al., 1997). In a study of 1003 Scandinavians (mostly White), it was found that CYP2D6 metabolism measured as the *O/N*-desmethylvenlafaxine ratio was significantly lower in carriers of *CYP2D6^∗^41* vs. *CYP2D6^∗^9–10* (Jukic et al., 2019). The annotated DPWG guideline states that for CYP2D6 poor (PM) and intermediate metabolizers (IM), select an alternative to venlafaxine or reduce the dose and monitor patient’s plasma metabolite level; for CYP2D6 ultrarapid metabolizers (UM), increase dose to 150% of the normal dose or select an alternative to venlafaxine.^19^

Duloxetine acts as a serotonin and noradrenaline reuptake inhibitor, and a weak dopamine reuptake inhibitor (e.g., in the frontal cortex).^20^ CYP1A2 and to a lesser extent CYP2D6 convert duloxetine into its main metabolites 4-hydroxy and 5-hydroxy duloxetine; activity (Knadler et al., 2011). CYP1A2 inducers including cigarette smoke therefore result in a reduction in duloxetine concentration (Augustin et al., 2018).

### Atomoxetine

Atomoxetine is a noradrenaline reuptake inhibitor used as second-line in the treatment of ADHD. It is metabolized mainly by CYP2D6 to 4-hydroxyatomoxetine and by CYP2C19 to *N*-desmethylatomoxetine, which is subsequently metabolized via CYP2D6 to *N*-desmethyl-4-hydroxyatomoxetine (Atomoxetine). Other enzymes (CYP1A2, CYP2B6, CYP2C19, CYP3A4, and CYP2E1) also contribute to the hydroxylation, with glucuronidation occurring subsequently (Yu et al., 2016).^21^ Atomoxetine may take 2-4 weeks for its full effect to be seen (Savill et al., 2015); peak concentrations have been associated with treatment efficacy and *CYP2D6* genotype has been associated with both peak concentration and half-life (e.g., exposure is on average 10-fold greater in CYP2D6 PMs; reviewed in the CPIC atomoxetine guidelines, Brown et al., 2019). Individuals homozygous for the *CYP2D6^∗^10* haplotype show a 5-fold higher peak atomoxetine concentration compared with individuals with at least one normal function haplotype; individuals heterozygous for the *CYP2D6^∗^10* also had higher atomoxetine exposure compared with CYP2D6 NMs (Cui et al., 2007; Matsui et al., 2012; Byeon et al., 2015).

The initiation dose for children and adolescents 0.5mg/kg/day. For UMs, NMs and IMs without a *CYP2D6^∗^10*, after three days an increase in dose to 1.2mg/kg/day is recommended. At the two week point, if there is neither efficacy nor adverse events, measurement of peak concentration 1–2 h after dose should be considered, and should this be less than 200 ng/ml, the dose may be increased until the concentration reaches 400 ng/ml. For those with an activity score of 0 (PMs), 0.5–1.0 (IMs including those with a *CYP2D6^∗^10*) the recommendation is that if there is neither efficacy nor adverse events by two weeks, to consider measuring plasma concentration 2–4 h (4 h for PMs) after dosing; if response is inadequate and the concentration is less than 200 ng/ml, the dose may be increased to approach 400 ng/ml; while if unacceptable side effects are present at any time, a reduction in dose should be considered. Of note, while the strength of the evidence for IMs, NMs, and UMs is moderate, for PMs, it is strong (Brown et al., 2019).

For adults, the starting dose is 40 mg/day. For UMs, NMs and IMs without a *CYP2D6^∗^10*, the dose should be increased to 80 mg/day after three days; if there is neither efficacy nor adverse events at two weeks, it is recommended to consider increasing the dose to 100 mg/day. After a further two weeks, if there is no clinical response, measurement of peak concentration 1–2 h after dose should be considered, and should this be less than 200 ng/ml, the dose may be increased until the concentration reaches 400 ng/ml. Doses above 100 mg/day may be needed to achieve target concentrations. For those with an activity score of 0, or 0.5–1.0 (IMs including those with a *CYP2D6^∗^10*), the recommendation is that if there is neither efficacy nor adverse events, at two weeks increase the dose to 80 mg/day. If resultant efficacy is inadequate, consideration should be given to measuring plasma concentration 2–4 h (4 h for PMs) after dosing; if the concentration is less than 200 ng/ml, the dose may be increased to approach 400 ng/ml; while if unacceptable side effects are present at any time, a reduction in dose should be considered.

To date one paper shows an association between *CYP2C19* and atomoxetine pharmacokinetics, with PMs showing higher atomoxetine concentration and half-life, and with correspondingly lower values for *N*-desmethylatomoxetine (Choi et al., 2014). Replication of this is required before any guidelines result.

## Conclusion

Many genetic variants in drug metabolizing enzymes and transporters have been shown to be relevant for psychiatry. Associations are strong enough to feature on drug labels and for prescribing guidelines based on such data (CPIC; DPWG). The International Society of Psychiatric Genetics recommends HLA-A and HLA-B testing prior to use of carbamazepine and oxcarbazepine, and suggests that genetic information for *CYP2C19* and *CYP2D6* would be likely to be most beneficial for individuals who have experienced insufficient efficacy or an adverse reaction to a previously tried antidepressant or antipsychotic (International Society for Psychiatric Genetics [ISPG], 2019). A range of (non-validated) commercial tests are currently available; however, there is variability in included genetic variants, methodology, and interpretation. This variability presents challenges for clinicians and other end users. Maruf et al. (2020) suggest the following should be considered: (a) whether or not the lab is accredited; (b) the relevance of the genetic variants to the medications of interest; (c) test logistics (such as turnaround time). With genes such as *CYP2D6* that are particularly challenging, a pragmatic approach may need to be taken: balancing a desire for a fast turnaround in a clinically accredited laboratory with a comprehensive coverage of all relevant functional variants.

While considerable progress has been made in determining reference samples by Gaedigk et al. (2019), what is still required is a consensus regarding the minimum set of informative variants in relevant genes that should be genotyped, methodologies for genotyping these efficiently and in a validated manner, and standardized interpretation with reporting algorithms and decision-support tools that can be integrated into electronic medical records. In addition, there has been relatively little work to date clinical associations with genetic variants in more than one gene (amitriptyline in Hicks et al., 2017; Greden et al., 2019, Aitchison et al., in submission). Depression was predicted to be responsible for the greatest global burden of disease by 2030 (Malhi and Mann, 2018) and in fact, given the evidence of increasing prevalence in association with the current viral pandemic (Holmes et al., 2020; Galea et al., 2020), this may be an underestimate. Depression is the mental health condition with the most prescribing guidelines in association with gene–drug pairs. Pharmacogenetically informed care has the potential to enhance treatment efficacy and reduce ADRs for this common disorder associated with not only the type of health burdens previously measured but also with a negative impact on outcomes from other health conditions ranging from cardiovascular to infectious diseases. Further, pharmacogenetics may not only reduce the risk of undesirable drug-drug interactions but may also in fact inform the utility of drug-drug interactions that may have a beneficial therapeutic effect – such as the induction of expression of *ABCB1* (Tian et al., 2005) (which may be associated with viral resistance). Significant ground in this area has been covered to date, but much remains to be covered. For example, *ABCB1* would appear to be the *CYP2D6* equivalent of drug transporters and is largely uncharted territory in terms of specific genotype–phenotype relationships by substrate binding including cooperativity, inhibition, and induction.

## Author Contributions

All authors contributed to manuscript drafting (each citation being reviewed by at least two authors), and approved the final version for publication.

## Conflict of Interest

KA is a member of the Pharmacogene Variation Consortium, Clinical Pharmacogenetics Implementation Consortium, has received two research grants in the last two years from Janssen Inc., Canada (fellowship grants for trainees) and provided consultancy services (unpaid) for HLS Therapeutics. The remaining authors declare that the research was conducted in the absence of any commercial or financial relationships that could be construed as a potential conflict of interest.

## Footnotes

1 https://www.pharmvar.org/gene/CYP1A2

2 https://www.pharmgkb.org/page/cyp2c19RefMaterials, accessed November 23, 2020.

3 https://www.pharmgkb.org/page/cyp2d6RefMaterials, accessed August 17, 2020.

4 https://www.pharmgkb.org/page/cyp2d6RefMaterials, accessed November 23, 2020.

5 https://www.pharmvar.org/gene/CYP3A5

6 https://www.pharmacogenomics.pha.ulaval.ca/ugt-alleles-nomenclature/, accessed May 17, 2020.

7 https://www.ncbi.nlm.nih.gov/snp, accessed July 12, 2020.

8 https://cpicpgx.org/prioritization/#cpicLevels

9 https://cpicpgx.org/genes-drugs/

10 http://upgx.eu/guidelines/, accessed May 1, 2020.

11 https://cpicpgx.org/genes-drugs/, accessed on September 23, 2020.

12 https://cpicpgx.org/prioritization-of-cpic-guidelines

13 https://www.knmp.nl/downloads/pharmacogenetic-recommendations-may-2020.pdf, accessed July 24, 2020.

14 https://cpicpgx.org/prioritization-of-cpic-guidelines

15 https://www.knmp.nl/downloads/pharmacogenetic-recommendations-may-2020.pdf, accessed July 23, 2020.

16 https://dailymed.nlm.nih.gov/dailymed/drugInfo.cfm?setid=217e52b5-3a7b-4bbe-af3c-b18de7d426c3, accessed May 1, 2020.

17 https://www.ema.europa.eu/documents/product-information/abilify-epar-product-information_en.pdf

18 https://www.accessdata.fda.gov/drugsatfda_docs/label/2006/018936s076lbl.pdf, accessed July 23, 2020.

19 https://www.pharmgkb.org/chemical/PA451866/guidelineAnnotation/PA166104968 PharmGKB, accessed May 18, 2020.

20 https://stahlonline.cambridge.org/prescribers_drug.jsf?page=9781316618134c40.html.therapeutics&name=Duloxetine&title=Therapeutics, accessed May 22, 2020.

21 https://www.pharmgkb.org/chemical/PA134688071, accessed on July 23, 2020.
